# Cobalt in end-of-life products in the EU, where does it end up? - The MaTrace approach

**DOI:** 10.1016/j.resconrec.2020.104842

**Published:** 2020-07

**Authors:** María Fernanda Godoy León, Gian Andrea Blengini, Jo Dewulf

**Affiliations:** a Research Group Sustainable Systems Engineering (STEN), Ghent University, Coupure Links 653, Ghent 9000, Belgium; b European Commission, Joint Research Centre (JRC), Directorate for Sustainable Resources, Land Resources Unit, Via E. Fermi 2749, Ispra, VA 21027, Italy; c Politecnico di Torino DIATI, Corso Duca degli Abruzzi 24, Torino, TO 10125, Italy

**Keywords:** Cobalt, Critical materials, MaTrace, Dynamic material flow analysis, Recycling

## Abstract

The use of cobalt has experienced a strong growth in the last decades. Due to its high economic importance and high supply risk, it has been classified as a critical raw material for the EU and other economies. Part of the EU's strategy is intended to secure its availability, through fostering its efficient use and recycling. The latter is affected by factors such as the amount of available end-of-life products, and their collection-to-recycling rate. A novel methodology to analyze the impact of these factors on the cobalt flows in society is the model MaTrace, which can track the fate of materials over time and across products. The MaTrace model was expanded, adapted, and applied to predict the fate of cobalt embedded in finished products in use in the EU, considering the underlying life cycle phases within the technosphere. Eleven scenarios were built, assessing different options in the implementation of relevant EU's policies. The flows were projected for a period of 25 years, starting in 2015. The results of the baseline scenario show that after 25 years, around 8% of the initial stock of cobalt stays in use, 3% is being hoarded by users, 28% has been exported, and 61% has been lost. The main contributors to the losses of the system are the non-selective collection of end-of-life products, and the export of end-of-life products, recycled cobalt and final products. The results of the scenarios show that higher collection-to-recycling rates and lower export could increase up to 50% the cobalt that stays in use.

## Graphical abstract

**Figure:**
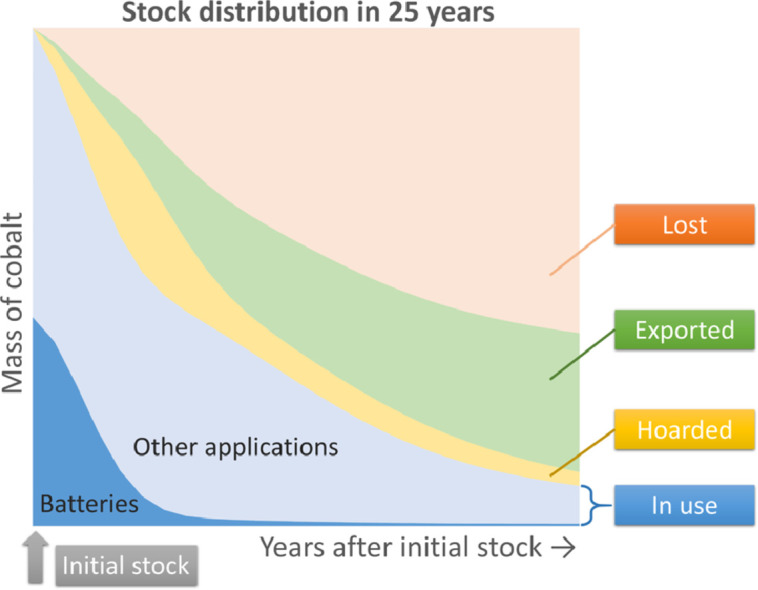

## 1 Introduction

Sustainable resource supply and management have become increasingly important, standing out as top priorities on the international political agenda. Accordingly, several initiatives have been launched in different parts of the world, e.g. the International Research Panel (IRP) of the United Nations, the Raw Materials Initiative of the European Union (EU) (European Commission, 2017a), the Critical Materials Strategy of the USA (USGS, 2018), the Critical Minerals Strategy of Australia (Commonwealth of Australia, 2019), and the Resource Securement Strategies of Japan (Hatayama and Tahara, 2014).

The EU, the USA, Australia, and Japan aim to secure their supply of certain materials that have been identified as critical/strategic, due to their economic importance and risk in their supply. A key aspect in this matter is the recycling of the materials, as a way to decrease the demand of virgin material, promoting at the same time security and a lower dependence on trade (IRP, 2017).

Several of these critical/strategic materials are metals, for which the recycled quantity depends on a number of factors, such as the efficiency of the pre-treatment and the recycling processes; and the amount, collection rate, and type of available end-of-life (EoL) products. There are certain products from which metals are not recovered due to economic and/or technological constraints, for example, products where the metal is present in very low concentrations (e.g. cobalt in printed circuit boards), or when their use is intentionally dissipative (e.g. metals in medicines or pesticides). Finally, metals can be functionally or non-functionally recycled. Through the former, the metal embedded in EoL products is separated, sorted, and sent back to raw material production processes, to be used again in the production of high-end products. Through the latter, the metal is collected and incorporated in an associated large-magnitude material stream, ending up in low-end products (also known as downcycling, where the original function is not required) or as a contaminant. While the metal is not dissipated into the environment, it is dissipated in the technosphere, as it is generally unfeasible to recover it from the large-magnitude stream (Buchert et al., 2009; Graedel et al., 2011; Zimmermann and Gößling-Reisemann, 2013; Zimmermann 2017).

Cobalt (Co) is one of the metals that has been classified as critical/strategic for the EU, the USA, Australia, and Japan. Its main producer country is the Democratic Republic of Congo, a country considered politically unstable (World Bank, 2019). It accounts for 68% of the global production, followed by the Philippines, Cuba and Russia (Darton Commodities Limited, 2018). In the last 20 years, its production and use have experienced a strong growth; its global refined production has increased in more than fourfold, from approximately 27,000 to 119,000 tons per year (BGS, 2019). This growth has been mainly driven by an increasing use of Co in the production of superalloys, catalysts, hard metals, and especially of rechargeable batteries (Shedd, 2010, 2017). The EU for instance used in 2012 nearly 20,000 tons of Co (20% of the total global consumption), of which around 50% was embedded in batteries (BIO by Deloitte, 2015). The recycling of Co has also grown in the last decades. Globally, it has been estimated that between 25 and 50% of the metal input to metal production corresponds to secondary Co (UNEP, 2011). In the EU, it amounts to around 35% (BIO by Deloitte, 2015). The main sources of secondary Co are batteries, catalysts, superalloys, and hard metals. However, within Europe, alloys containing Co are predominantly downcycled into stainless steel (European Commission, 2017b). In addition, there are products from which Co cannot be recovered, for example pigments, glass, and paints (RPA, 2012). The Co cycle has been studied globally and regionally, fully or partially addressing the supply, demand, stock and flows of the metal (National Research Council, 1983; OTA, 1985; Shedd, 1993; Harper et al., 2012; RPA, 2012; BIO by Deloitte, 2015; Alves Dias et al., 2018; European Commission, 2018a; Godoy León and Dewulf, 2020). However, these studies have been developed for a single year (some of them already outdated reports), not capturing the dynamics of the flows and stocks over several years.

From the previous paragraphs, it is clear that a good understanding of the societal metabolism of metals, and in particular of Co, is key to enhance their recycling and sustainable management, keeping them in the circular economy. With this purpose, different studies and models have been developed in the context of Material Flow Analysis (MFA), aiming to a better understanding of metal recycling. Particular attention has been given to dynamic MFA (dMFA) (Elshkaki et al., 2005; Hatayama et al., 2010; Milford et al., 2013; Müller et al., 2014; Wang et al., 2017), which has been used to model the impact of stock growth and the application of different recycling technologies on the material cycles. One example is the model MaTrace (Nakamura et al., 2014), which is an input-output dMFA model that can track the fate of materials (a specific initial stock) over time and across products in open-loop recycling. It was implemented in Japan to track the fate of steel that was initially part of a car, over a 100-year period. MaTrace is the first IO-based dynamic MFA model that explicitly considers the losses incurred during the conversion processes. However, there are aspects that it does not take into account, e.g. the hibernation or hoarding of products at the end of their service, and the export of material as a separate stream. Two other models were derived from MaTrace: MaTrace Global (Pauliuk et al., 2017) and MaTrace-Alloy (Nakamura et al., 2017). The former is a multiregional extension of MaTrace with a global scope, which was used to study steel flows in twenty-five regions of the world. The latter was used to track element flows instead of material (chromium and nickel embedded in steel). To the best of the authors’ knowledge, the model MaTrace has not been applied to model flows in Europe or the EU, nor to track any material other than steel.

The focus of this research is to improve the understanding of the Co cycle in the EU, assessing its circularity and the dynamics of its behavior over a longer time period. The objective is to predict the fate of Co embedded in finished products to be used in the EU, considering the underlying life cycle phases within the technosphere. By using an expanded and adapted version of MaTrace, these flows are forecasted for a period of 25 years, starting in 2015. New aspects were added to the model, related to the hoarding of products (dead storage of a product that has reached the end of its use) and the export of material. Important data for the modeling are specific parameters related to production, manufacturing, use, end-of-life, and recycling per category of products, e.g. lifetime of products, collection rates, and recycling efficiency. The model quantifies the amount of Co that stays in high-end products during the assessment period, together with the amount hoarded by users; the amount exported; and the losses due to non-selective collection, downcycling, and inefficiencies of the pre-treatment, recycling, and production processes.

## 2 Methodology

### 2.1 Model MaTrace

The model MaTrace was the basis of this research. This model tracks the fate of material initially embedded in defined categories of products, in a specific region, over time, and across products. It is important to indicate that the model allows tracking only an initial stock of material within the system under study, but that it does not track material that enters the system later on.

Two new features were incorporated into the model, the hoarding of end-of-service (EoS) products and a separate stream for the export. Hoarding (also known as hibernation) refers to the dead storage of a product that is no longer in use, e.g. an old mobile phone kept in the attic (Wilson et al., 2017). The material is not lost but neither available for recycling. The export stream considers the export of recycled material, manufactured products, and end-of life (EoL) products. It was included to estimate the economic losses for the region.

Another difference with the MaTrace from Nakamura et al. (2014) is the distinction between functional recycling and non-functional recycling. In their paper, the model was developed for open-loop recycling, considering different scrap quality. In this research, a distinction was made between the material that stays in high-end products through functional recycling, and the material that goes to low-end products, i.e. the downcycled material, through non-functional recycling.

**Fig. 1:**
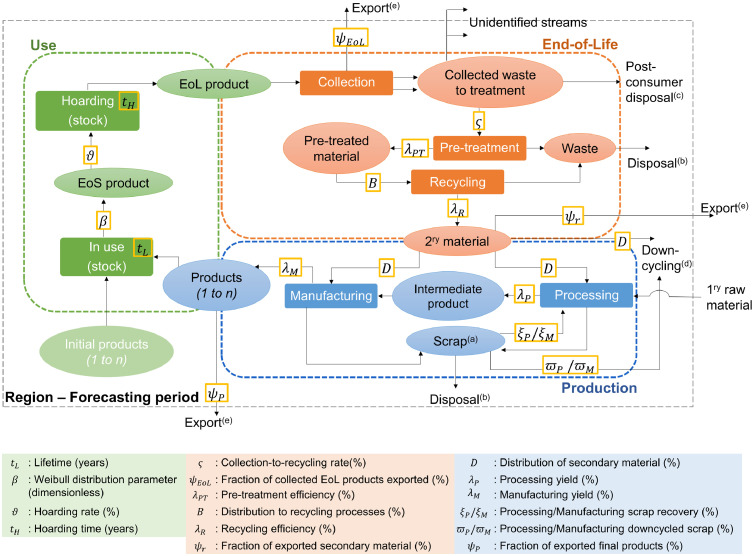
Identified life cycle phases (color dashed rectangles) and parameters (yellow rectangles) for each one. Ovals represent materials, products or waste; solid rectangles represent processes or sub-phases of the life cycle, black dashed rectangle represents the system boundaries (primary raw material is out of scope). The percentages are weight percentages. EoL: End-of-Life, EoS: End-of-Service. (a) New and prompt scrap. (b) Physical losses through inefficiencies of the processes (*L_I_*). (c) Physical losses through post-consumer disposal (*V*). (d) Physical losses through downcycling (*D_T_*). (e) Economic losses through export (*E_T_*). Adapted from Godoy León and Dewulf (2020).

The studied system is depicted in Fig. 1, which was adapted from our previous research (Godoy León and Dewulf, 2020). The definition of the parameters can also be found in that publication. Following, the application of the model and the new features are described per life cycle phase. In addition, the losses and stocks of the system are defined. The nomenclature of the parameters, units, and the complete set of equations are available in SI.

#### 2.1.1 Use phase

This phase comprises two sub-phases: In use and Hoarding. As in the original model, the Weibull distribution was used to model the lifetime of the considered product. By the use of the distribution, the amount of EoS products was calculated. A new feature was added to consider the stock of EoS products, for which the hoarding rate and the hoarding time of the different products (1 to *n* categories of products) were required. The amount of hoarded product *i* in year *t* (*K_i_*(*t*)) was calculated as:

(1) $$K_{i}\left(t\right)=\vartheta_{i}z_{i}\left(t\right)\left(i:1,\cdots ,n;t:1,2,\cdots \right)$$

Where ϑ_*i*_ is the hoarding rate (%) of product *i*, and *z_i_*(*t*) is the available EoS product *i* in year *t*.

It is important to indicate that for the sake of simplicity and given limited availability of data, it was assumed that there is no re-use of products in the region. While there are studies that assess the reuse and extension of lifetimes of products (Thiébaud et al., 2017, 2018; Glӧser-Chahoud et al., 2019), these normally focus on a limited range of applications. This assumption means that EoS products become EoL products after their storage period. EoS products become immediately EoL products when the hoarding time is equal to zero. It is also important to note that the phase In use refers exclusively to material that is embedded in high-end products.

#### 2.1.2 EoL phase

The EoL phase considers the collection, pre-treatment, and recycling of EoL products. The collection consists of the amount of products that are immediately disposed at their end of life, and of the amount of products released after the hoarding period:

(2) $$C_{i}\left(t\right)=\left(1-\vartheta_{i}\right)z_{i}\left(t\right)$$

(3) $$R_{p,i}\left(t\right)=K_{i}\left(t-p\right)$$

(4) $$C_{T,i}\left(t\right)=C_{i}\left(t\right)+R_{p,i}\left(t\right)$$

Where *C_i_*(*t*) is the amount of collected EoL product *i* in year *t* (which was immediately collected at the end of its service period), *R*_*p,*__*i*_(*t*) is the amount of EoL product *i* released in year *t* after *p* years of hoarding, and *C*_*T,*__*i*_(*t*) is the total amount of collected EoL product *i* in year *t*.

The collected EoL products follow one of two paths: export (*E*_*EoL*__*,*__*i*_) or treatment (*U_i_*(*t*)). Treatment considers disposal (called post-consumer disposal) (*V*_*i*_) or recycling (*G*_*i*_). These streams come from two type of collection: non-selective and selective (in Fig. 1, see two arrows between ‘Collection’ and ‘Collected waste to treatment’). The former ends up with the disposal of the EoL product and the latter with its recycling.

(5) $$E_{\mathrm{EoL},i}\left(t\right)=\psi_{\mathrm{EoL},i}C_{T,i}\left(t\right)$$

(6) $$U_{i}\left(t\right)=\left(1-\psi_{\mathrm{EoL},i}\right)C_{T,i}\left(t\right)$$

(7) $$G_{i}\left(t\right)=\varsigma_{i}U_{i}\left(t\right)$$

(8) $$V_{i}\left(t\right)=\left(1-\varsigma_{i}\right)U_{i}\left(t\right)$$

Where *ψ*_*EoL,i*_ is the percentage of collected EoL product *i* that is exported, and *ς*_*i*_ is the percentage of collected EoL product *i* that goes to recycling facilities (collection-to-recycling rate).

As it is possible to observe in Fig. 1, the collected waste can also move in the technosphere through unidentified streams. These correspond to gaps between the reported quantities of recycled waste and the collected waste. In case these streams are considered, these gaps have to be taken into account when calculating the amount that is recycled and the amount that is disposed.

Prior to recycling, the collected EoL products are pre-treated. The pre-treatment consists of separation, sorting, physical processing, chemical processing, or a combination of these. The recovered material is afterwards recycled. As in the original model, an allocation matrix *B* was used. This matrix consists of the distribution of scrap to the recycling processes (recovered material to be used in high-end products, functional recycling) and to downcycling processes (recovered material to be used in low-end products, non-functional recycling).

A separate stream was established for the export of functionally recycled material, considered as a loss for the system.

#### 2.1.3 Production phase

The secondary material obtained from the recycling processes that stays in the region is used in the production of new products. The distribution of the material among the different product categories is given by matrix *D*. To establish this matrix, Nakamura et al. (2014) made use of a monetary input-output table (MIOT) which was transformed into a physical input-output table (PIOT). The use of these tables is understood as an advantage in terms of flexibility and accuracy of the estimation. However, it also requires highly detailed information (high level of resolution of the tables) that is not always available. This is the case for Co, for which the existing IOTs do not possess the required resolution level. For this reason, in this study matrix *D* was established based on literature. The same reasoning applies to the estimation of vector *λ* (yield ratio of final product), which was estimated considering the processing yield of intermediates (alloys or precursors), and the manufacturing yield of final products.

The downcycled material during the processing (*D*_*p,*__*i*_) and manufacturing (*D*_*m,*__*i*_) phases is also considered in the adapted model. Finally, in the original model the use included both domestic and export uses. Here, a clear distinction for the export of final products was made.

The model assumes that the production and use of a product occurs in the same year.

#### 2.1.4 Losses

The adapted model considers two types of losses: physical loss and economic loss. The former corresponds to the material that is dissipated into the environment and the technosphere. It includes the losses that arise from the inefficiencies of the involved processes (processing, manufacturing, pre-treatment, and recycling), the downcycled scrap, and the non-selective collection. The latter corresponds to the material that leaves the system through export. The export is not a loss for the whole society across the world, but only for the studied region. Both types of losses occur in the Production and EoL phases (see Fig. 1).

Losses from the Use phase may occur when the use of the material is intentionally dissipative. For instance, material embedded in pesticides and medicines is immediately dissipated at the moment of use. In other cases, the material is lost at the end of the product's life, for example, pigments in ceramics. In this model, the second approach was considered; hence, there are no losses from the Use phase.

The total loss from process inefficiencies (*L_I_*) is given by:

(9) $$L_{I}\left(t\right)=\sum_{i=1}^{n}\left(L_{P,i}\left(t\right)+L_{M,i}\left(t\right)+L_{\mathrm{PT},i}\left(t\right)+L_{r,i}\left(t\right)\right)$$

Where *L*_*P,*__*i*_(*t*), *L*_*M,*__*i*_(*t*), *L*_*PT,*__*i*_(*t*), *L*_r__*,*__*i*_(*t*) are the losses of metal in year *t* due to inefficiencies of the processing, manufacturing, pre-treatment, and recycling processes related to product *i*, respectively. The symbol *n* corresponds to the total number of categories of products.

The total amount of downcycled material during production and recycling (*D_T_*) is given by:

(10) $$D_{T}\left(t\right)=\sum_{i=1}^{n}\left(D_{P,i}\left(t\right)+D_{M,i}\left(t\right)+D_{r,i}\left(t\right)\right)$$

Where *D*_*r,*__*i*_(*t*) is the downcycled material of the recycling sub-phase, of product *i* in year *t*.

The loss by export is given by the export of EoL products (*E*_*EoL*__*,*__*i*_), the export of functionally recycled material (*E*_*r*__*,*__*R*_), and the export of final products (*E*_*P*__*,*__*i*_). Then, the total export in year *t* (*E_T_*(*t*)) is:

(11) $$E_{T}\left(t\right)=\sum_{i=1}^{n}E_{\mathrm{EoL},i}\left(t\right)+\sum_{R=1}^{n_{R}}E_{r,R}\left(t\right)+\sum_{i=1}^{n}E_{P,i}\left(t\right)$$

Where *n_R_* is the number of type of recycled material.

Finally, the total loss for the system is:

(12) $$L_{T}\left(t\right)=L_{I}\left(t\right)+\sum_{i=1}^{n}V_{i}\left(t\right)+D_{T}\left(t\right)+E_{T}\left(t\right)$$

The aggregated losses (addition of the losses from all applications) (*L_a_*) occurring in year *t* are calculated as:

(13) $$L_{a}\left(t\right)=\sum_{i=1}^{n}\left(z_{i}\left(t\right)+R_{p,i}\left(t\right)-x_{\mathrm{EU},i}\left(t\right)\right)$$

Where *x*_*EU,*__*i*_(*t*) is the amount of product *i* in use in the region in year *t*.

The aggregated losses have to be equal to:

(14) $$L_{T}\left(t\right)+\sum_{i=1}^{n}K_{i}\left(t\right)$$

#### 2.1.5 Stock

The stock of product *i* being used in the region in year *t* is:

(15) $${\bar{x}}_{EU,i}\left(t\right)=\sum_{r=0}^{t}\left(1-\sum_{p=0}^{t-r}E_{i}\left(p\right)\right)x_{EU,i}\left(r\right)$$

Where *E_i_*(*p*) is the fraction of discarded product *i* after *p* years of use, and *r ≤ t.*

The stock of losses is given by:

(16) $${\bar{L}}_{T}\left(t\right)=\sum_{r=0}^{t}L_{T}\left(r\right)$$

Then, by mass conservation:

(17) $$\sum_{i=1}^{n}{\bar{x}}_{\mathrm{EU},i}\left(t\right)+{\bar{L}}_{T}\left(t\right)-\bar{R_{p}}\left(t\right)=\sum_{i=1}^{n}x_{\mathrm{EU},i}\left(0\right)$$

With *x*_*EU,*__*i*_(0) being the initial distribution of material in the region, and $\bar{R}p$ the stock of released hoarded products:

(18) $$\bar{R_{p}}\left(t\right)=\sum_{r=0}^{t}\sum_{i=1}^{n}R_{p,i}\left(r\right)$$

### 2.2 Product categories, recycling processes, and data

Based on our previous research (Godoy León and Dewulf, 2020) seven high-end product categories of Co were studied: batteries, catalysts, dissipative uses (e.g. pigments), hard metals, magnets, superalloys, and other metallic uses (e.g. tool steels and semi-conductors). The category batteries was divided in two sub-categories: portable batteries and mobility batteries. The main focus was Li-ion batteries, as it is the most widely used among Co batteries (Cobalt Institute, 2018b). In the case of catalysts, three sub-categories were studied: for hydroprocessing, for hydroformylation, and for the production of polyester (PET) precursors. A description of these product categories can be found in Godoy León and Dewulf (2020).

Matrix *B* was established considering three recycling processes: a chemical process for functional recycling, obtaining Co metal or a Co compound; the Zn process for functional recycling, obtaining W-Co powder; and a downcycling process (non-functional recycling). The products recycled by the first process are batteries, catalysts, hard metals, and superalloys. The Zn process is only used to recycle hard metals, and the downcycling process is used in the recycling of catalysts, magnets, other metallic uses, and superalloys. Given the purpose of this study, the recycling processes were not analysed in detail, as the model only requires the efficiency of the whole phase (the same applies for the pre-treatment). However, it is important to keep in mind that these phases consist of sub-processes. For example, in the case of Li-ion batteries, the pre-treatment includes mechanical separation (applying crushing, shredding, sorting and sieving steps, magnetic separation and air separation methods), a thermal process (heating the samples at 150–500 °C), a dissolution process (using solvents), and physical-chemical methods (using high-energy ball milling to induce physical and chemical changes of active materials). Regarding the recycling processes, these have been classified as hydrometallurgy-dominant methods, where valuable metals are separated by leaching, precipitation and solvent extraction; pyrometallurgy-dominant methods, where high temperature (higher than 1400 °C) is used to enhance the physicochemical separation of valuable metals; and mild recycling methods, where hydrometallurgy-dominant and pyrometallurgy-dominant methods are integrated (Zhang et al., 2014; Liu et al., 2019). Clearly, a number of steps are required for the recycling of Li-ion batteries, and different combinations of processes can be applied.

For the estimation of matrix *D*, it was necessary to determine the distribution of secondary material among Co products. According to literature and information obtained through personal contacts (Shedd, 2004; Sommer et al., 2015; Cobalt Institute, 2018a; Umicore, 2018), functionally recycled Co can be used in the production of any product. Therefore, its distribution is given by the demand of Co in the region (see Table 1). The W-Co powder is used in the manufacturing of new hard metals (Katiyar et al., 2014; Freemantle and Sacks, 2015; Kurylak et al., 2016). In the case of downcycling, it was considered that the recovered metallic stream is used in the production of stainless steel (Akcil et al., 2015; European Commission, 2017b). The values of matrix *B* and *D* are available in SI.

**Table 1:** Initial distribution in 2015 of Co among ten high-end product (sub)categories (*x_EU_*(0)) in use in the EU, and destination of the secondary metal (functionally recycled through the chemical process) for high-end products production in the BAU scenario. See calculation in SI.

Product category	Initial distribution of Co in 2015 *x_EU_*(0) (wt%)	Destination of recycled Co from chemical process 2016–2040 (wt%)
Portable batteries	41.2	0.0
Mobility batteries	1.2	2.1
Hydroprocessing catalysts	2.0	2.7
Hydroformylation catalysts	0.3	0.5
PET precursors catalysts	1.7	2.4
Dissipative uses	9.0	16.7
Hard metals	23.0	31.3
Magnets	0.6	1.7
Other metallic uses	0.2	0.3
Superalloys	20.8	42.3

The data related to the parameters of the model were likewise acquired from Godoy León and Dewulf (2020). In that publication, data related to Co flows were gathered and analysed in function of their quality. In this work, the values with the highest quality were used. The values of parameters not considered in that study, such as the export percentage of final products and recycled material were obtained from literature. A number of parameters were characterized by multiple values. In this case, the average value was used. Other parameters were assumed due to data gaps. These assumptions were made based on the values of the same parameter of other products of Co. The data and assumptions used per product category are described in SI.

### 2.3 System boundaries and baseline scenario

The original model was applied to track the fate of steel originally embedded in a car (single product), starting to be used in year 0. In this research, the model was implemented to track the flows of Co initially embedded in ten high-end (sub)categories of products. This initial stock considers the Co embedded in finished products, both produced in and imported to the EU in 2015, which will be used in the same year. It was defined as ‘2015 EU-Co in finished products’. Table 1 lists the initial distribution of the metal among these products (*x_EU_*(0)).

The initial year of the study was 2015. The model was applied to forecast the fate of Co in the EU over 25 years (until 2040). It was considered that this time span is appropriate to measure circularity of materials in function of the current generation, being the longest period that allows extrapolation of current technologies and practices reasonably. Beyond 25 years, all extrapolations are considered too speculative (e.g. Schulze et al. (2020)) used 25 years in their work as short to middle term).

The baseline scenario consists of a business as usual (BAU) scenario, in which the value of the parameters is constant in time.

### 2.4 Sensitivity analysis

A sensitivity analysis was performed to assess the extent to which the results are affected by changes in the value of the parameters. Two groups of parameters were analysed: parameters presenting multiple values, and parameters for which one single value was assumed. For the first group, the sensitivity analysis was developed using the minimum and the maximum found values. For the second group, the analysis was developed considering ± 10, 20 and 30% of the assumed value. In the case that lifetimes or hoarding times were assumed, the analysis was developed considering ± 1, 2 and 3 years compared to the assumed value.

Considering both groups, 56 of the 242 parameters of the model were analysed. The analysis was developed changing one parameter at the time, and leaving the rest as in the baseline scenario. It was studied how the value of each parameter affects the distribution of Co, compared to the BAU scenario. Three forms of distribution were analysed: in use, exported, and physically lost. For each distribution form, the percentage of variation was calculated year by year. Then the average value was calculated for the complete period. Finally, the parameters showing an impact of at least 15% compared to the BAU scenario were selected for the analysis.

### 2.5 Other scenarios and indicators

Next to the BAU scenario, where constant parameters were considered, other five main scenarios were built to assess potential impacts of policy in the EU. These scenarios were built considering changes in the collection-to-recycling rates, in the demand of Co for the production of mobility batteries, and in the export. The rest of the parameters were kept as in the BAU scenario. Table 2 summarizes the changes per scenario.

**Table 2:** Applied changes per scenario. Nine (sub)scenarios were analysed, sub-scenarios Collection rate-policy(5–10–20) and Collection rate-max(5–10–20) with different increase of the collection-to-recycling rate, plus scenarios 4 to 6. BAU: business as usual. ^a^ The collection-to-recycling rate increases to 45% for portable batteries, and 85% for magnets and other metallic uses; afterwards it remains constant. ^b^ The collection-to-recycling rate increases to 100% for all applications (except dissipative uses). ^c^ The demand increases until 2030, afterwards it remains constant.

Scenario number	Scenario name	Parameter or aspect of the model		
		Collection-to-recycling rate	Demand for production of mobility batteries	Export of EoL products and recycled Co
2	Collection rate-policy ^a^	Increase 5, 10 or 20% per year	BAU	BAU
3	Collection rate-max ^b^	Increase 5, 10 or 20% per year	BAU	BAU
4	Increase production- mobility battery ^c^	BAU	Increase 24% per year	BAU
5	No export	BAU	BAU	Zero
6	Combined ^b, c^	Increase 5% per year	Increase 24% per year	Zero

Following, a description of each scenario is given. The calculations related to each scenario are available in SI.

#### Scenario 2. Collection rate-policy: collections-to-recycling rates according to EU's Directives

This scenario was built considering an increase of the collection-to-recycling rates of portable batteries, magnets, and other metallic uses, to the levels indicated in the EU's Batteries Directive and the WEEE Directive: 45% for batteries, and 85% for WEEE (European Commission, 2006, European Commission, 2012). Three sub-scenarios were built: ‘Collection rate-policy5’, ‘Collection rate-policy10’, and ‘Collection rate-policy20’; considering a constant increase of 5, 10, and 20% per year. After reaching the aforementioned levels, the collection-to-recycling rates remained constant. The increase of the collection-to-recycling rates was compensated by a proportional and equally shared decrease of the non-selective collection and hoarding rates.

#### Scenario 3. Collection rate-max: collection-to-recycling rates increase to 100%

This scenario considers the ideal case in which all products are collected to be recycled in 100% (except for dissipative uses). Three sub-scenarios were built: ‘Collection rate-max5’, ‘Collection rate-max10’, and ‘Collection rate-max20’; considering a constant increase of 5, 10, and 20% per year, until reaching 100% of collection. As in the previous scenario, the increase of the collection-to-recycling rates was compensated by a proportional and equally shared decrease of the non-selective collection rates and the hoarding rates.

#### Scenario 4. Increase production-mobility battery: increase of the production of mobility batteries

The BAU scenario considers a constant demand of Co among the different categories of products during the studied period. However, it is expected that the production of mobility batteries will increase substantially in the coming years. This scenario was built to address this growth. It was established based on the EU Batteries Alliance, which aims to create a competitive battery cell manufacturing in Europe. According to the European Commission, EU's Co demand for hybrid and electric vehicles will increase from 510 tonnes in 2015 to almost 13,000 tonnes in 2030 (European Commission, 2018b). Based on this information, the demand of mobility batteries was forecasted between 2015 and 2030, assuming that after 2030 the share of the demand remains constant. The increase of the demand was compensated by a proportional and equally shared decrease of the demand of the other products.

#### Scenario 5. No export: no export of EoL products and recycled material

This scenario reflects the case in which there is no export of EoL products and recycled Co throughout the studied period. In this way, the Co is longer available in the EU. In this scenario, the export of final products is still considered.

#### Scenario 6. Combined: combination of scenarios

Combination of scenarios ‘Collection rate-max5’, ‘Increase production-mobility battery’, and ‘No export’, to analyze the simultaneous effect of the change of the collection rates, the mobility battery production, and the export.

Each scenario was compared to the BAU scenario. The comparison was made based on an indicator presented by Klose and Pauliuk (n.d.), *p_i_*(*t*), which is the percentage of Co in form *i* after *t* years (with *i*: I, in use; H, hoarded, E, exported; L, lost). The percentage of each Co distribution form of each scenario for a given year was compared to the corresponding percentage of the BAU scenario. This comparison was termed *Q_i_*(*t*):

(19) $$Q_{i}\left(t\right)=\frac{p_{i,\mathrm{BAU}}\left(t\right)-p_{i,X}\left(t\right)}{p_{i,\mathrm{BAU}}\left(t\right)}\;\;\;\left(X:\left(\mathrm{sub}\right)\mathrm{scen}\mathrm{arios}2\mathrm{to}6\right)$$

Another indicator estimated for each scenario (including the BAU scenario) was the half-life time of Co, termed π. This indicator refers to the years that Co takes to be present in less than 50% in use.

## 3 Results and discussion

### 3.1 Baseline scenario

**Fig. 2:**
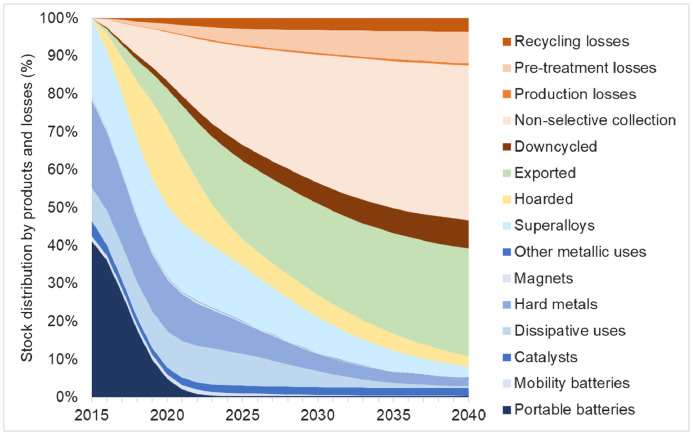
Transition in the composition of the stock of Co in products, hoarded products, export, and physical losses. Due to their low contribution to the stocks, the three type of catalysts were aggregated in a single category (catalysts), and the processing losses and manufacturing losses were added in production losses.

Fig. 2 presents the transition in the composition of the stock of Co in high-end products, hoarded products, export, and physical losses over time for the BAU scenario. It is observed that the main product categories of Co are dissipative uses, hard metals, and superalloys. This is given by the initial distribution of Co in the high-end products, and by the destination of the secondary material used for their production. Even though, initially the highest amount of Co is present in portable batteries, in 7 years it decreases to less than 1% of the initial stock. Currently, there is no production of portable batteries in the EU (EBRA, 2018); therefore, after 7 years, which correspond to the lifetime plus the hoarding time of portable batteries, the ‘2015 EU-Co in finished products’ is nearly not present in this type of devices.

The baseline scenario shows that after 10 years only 35% of the ‘2015 EU-Co in finished products’ stays in use, 7% is hoarded, 21% has been exported from the EU, and 38% has been physically lost. Of these losses, the biggest contribution comes from the non-selective collection (26%), which accounts for 69% of the total losses. After 25 years, only 8% is still in use, 3% is being hoarded, 28% has been exported, and 61% has been physically lost. The half-life time of Co in use (indicator π) is 6 years.

In the BAU scenario, unidentified streams were not considered, assuming that they follow the same distribution as identified streams. To assess to which extent these flows could possibly affect the results, the model was run considering unidentified streams of WEEE (related to magnets and other metallic uses) and Li-ion batteries (Huisman et al., 2017). It was found that around 17% of the ‘2015 EU-Co in finished products’ ends up in unidentified streams (see calculations in SI). This is mainly given by the high percentage of unidentified streams of portable Li-ion batteries (42% collection-to-recycling rate, 17% disposal rate, and 41% gap).

### 3.2 Sensitivity analysis

**Fig. 3:**
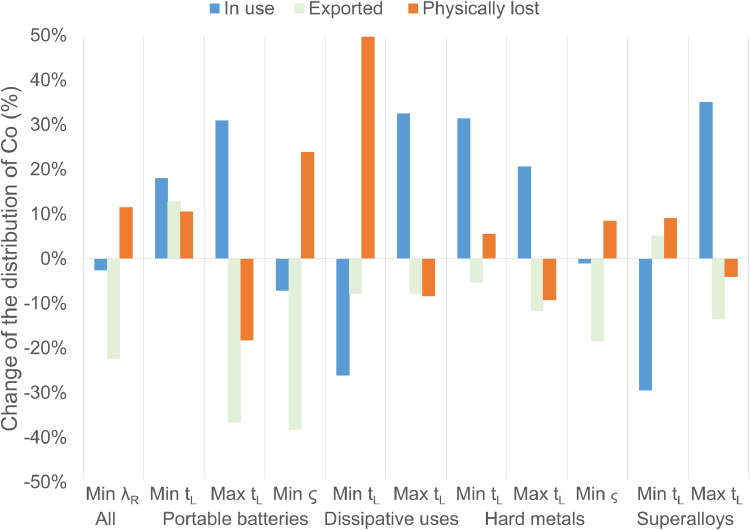
Effect of 11 parameters (per application) on the distribution of Co: in use, exported, and physically lost, when they take their minimum or maximum value. The results present the average of the annual change from 2015 to 2040, in comparison to the baseline scenario (BAU). Min: minimum value of the parameter, Max: maximum value of the parameter, All: all product categories, λ_R_: recycling efficiency, t_L_: lifetime, ς: collection-to-recycling rate.

For the sensitivity analysis, 56 parameters were analysed. Thirty-seven correspond to parameters with multiple values, and 19 correspond to assumed single values. Of the 37 parameters with multiple values, 11 present an effect (in absolute terms) of over 15% change in at least one of the three analysed forms of Co distribution: in use, exported, and physically lost. This can be observed in Fig. 3, which presents the change of each form of distribution compared to the BAU scenario. For each form of distribution, the change was calculated per year (from 2015 to 2040), and then an average (along the study period) was calculated.

Changing the value of these parameters has diverse effects. For example, it is clear that a lower value of the recycling efficiency produces an increase of the physical losses, due to an increase of the recycling losses. At the same time, a lower efficiency generates less recycled Co, therefore, less available Co to be exported as secondary material and embedded in final products. The same logic applies to the effect of using the minimum value of the collection-to-recycling rate of portable batteries and hard metals. Due to a lower selective collection, there is a higher physical loss due to post-consumer disposal, less Co available to be recycled, and consequently, less available Co to be exported.

Related to the effect of the lifetime, it is observed that the minimum values generate an increase of the physical losses, and the maximum values a decrease of them. At a lower lifetime, more of the ‘2015 EU-Co in finished products’ is available in EoL products to be collected, recycled, processed, manufactured, and exported. Hence, more Co is lost due to the non-selective collection, downcycling, and the inefficiencies of the processes. Clearly, a higher lifetime has the opposite effect. For the Co in use and the export, the effect of the lifetime varies from product to product. Using the maximum values increases the amount that stays in use, and decreases the amount exported. However, when using the minimum values there are no clear trends. The minimum of the lifetime of portable batteries and hard metals increases the amount of Co in use, and the minimum of the lifetime of dissipative uses and superalloys decreases it. The export in turn increases with the minimum of the lifetime of portable batteries and superalloys, and decreases with the minimum of the lifetime of dissipative uses and hard metals. The reason of this is that the amount of Co in use does not only depend on the lifetime of the products, but also on how the secondary material is distributed among them, which also affects the amount of Co exported. Portable batteries and hard metals have a shorter lifetime than dissipative uses and superalloys (3.4 and 6.5 years compared to 13 and 14 years, respectively). In addition, these two last applications are two of the main demands of Co for manufacturing in the EU. Thus, portable batteries and hard metals with lower lifetimes (1 year each) produce more Co available in a shorter period, which will be mainly used for the production of superalloys and dissipative uses, making that Co stays longer in use compared to the original case.

Clearly, the results are more or less sensitive depending on the assessed parameter. However, it is important to point out that this analysis does not indicate to which parameters the model is more sensitive, but which of the parameters with multiple values are critical in this specific case. This depends, among other things, on how different are the minimum and maximum values from each other (e.g. the lifetime of dissipative uses can be 1 year or between 5 and 25 years). In order to obtain more reliable results, it is imperative to establish a single and sound value for these parameters.

Of the 19 parameters for which a value was assumed, two present an effect (in absolute terms) of more than 15% in at least one of the forms of distribution: the hoarding time of magnets and the hoarding time of other metallic uses. These only affect the amount hoarded (higher hoarding time, higher amount hoarded).

### 3.3 Scenarios and indicators

Nine (sub)scenarios were analysed. Three of them, sub-scenarios ‘Collection rate-policy5’, ‘Collection rate-policy10’, and scenario ‘Increase production-mobility battery’ did not present substantial differences compared to the BAU scenario. In the case of sub-scenarios ‘Collection rate-policy5’ and ‘Collection rate-policy10’, the increase of the collection-to-recycling rates occurs too slowly to have a significant impact. Interesting is to observe that scenario ‘Increase production-mobility battery’ do not increase the amount of Co that stays in mobility batteries. The reason of this is that, even though the Co demand for this product increases noteworthy, there are still other products with a bigger demand. In addition, the amount of ‘2015 EU-Co in finished products’ available to be new products is very little. This is due to one of the limitations of the model, which does not capture the Co that enters to the economy after 2015, e.g. the 2020-Co.

**Fig. 4:**
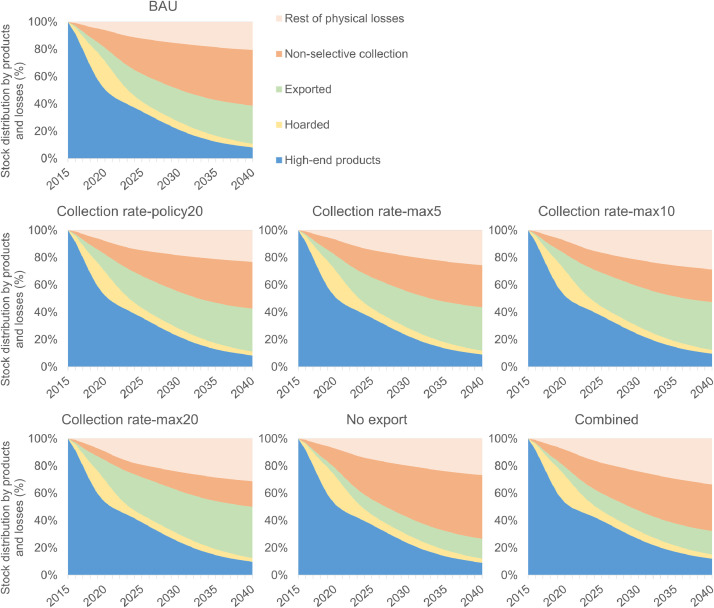
Transition in the composition of the stock of Co in products, hoarded products, export, non-selective collection, and the rest of the physical losses. BAU: baseline scenario. Collection rate-policy20: increase of collection-to-recycling rates according to EU's Directive; 20% per year. Collection rate-max5 to Collection rate-max20: increase of collection-to-recycling rates to 100%; 5, 10, and 20% per year. No export: No export of EoL products and recycled material. Combined: Combination of scenarios ‘Collection rate-max5’, ‘Increase production-mobility battery’, and ‘No export’. High-end products include the seven high-end products of Co. Rest of physical losses include all physical losses except for non-selective collection.

The transition in the composition of the stock of Co of the other six scenarios is presented in Fig. 4. For a better visualization of the results, the Co in use of the different products was aggregated in a single category, called high-end products. In the same way, the physical losses were aggregated in a single category, called rest of physical losses. This category includes all physical losses, except for the non-selective collection, which is showed separately due to its high contribution to the losses. The detailed graphs are shown in SI.

Sub-scenarios ‘Collection rate-policy20’, ‘Collection rate-max5’, ‘Collection rate-max10’, and ‘Collection rate-max20’, based on an increase of the collection-to-recycling rates, present a clear decrease of the losses by non-selective collection, compensated by an increase of the export and of the rest of the physical losses. The latter is mainly due to an increase of the downcycled material. Scenario ‘No export’, which considers no export of EoL products and recycled material, is characterized by an increase of the losses by non-selective collection and of the rest of the physical losses (mainly of the downcycled material), and an expected decrease of the export. Finally, scenario ‘Combined’ that is a combination of scenarios ‘Collection rate-max5’, ‘Increase production-mobility battery’, and ‘No export’, is characterized by an increase of Co in high-end products and downcycled, and by a decrease of the losses by non-selective collection and of the export. It is important to indicate that scenarios ‘No export’ and ‘Combined’ still present some exported Co, due to the export of final products.

**Fig. 5:**
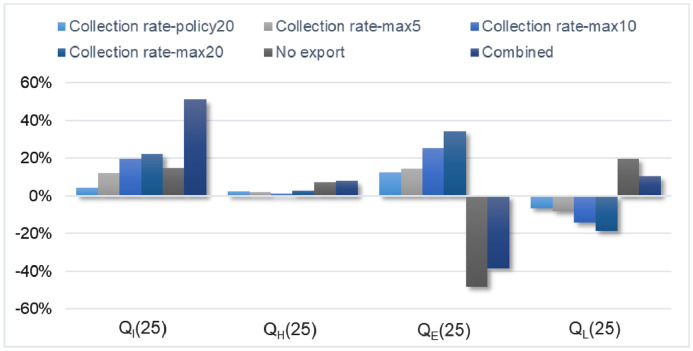
Comparison of *Q_i_*(*t*) for t equal to 25 years, with i: I: in use, H: hoarded, E: exported, L: lost (referring to physical losses). Relative to the baseline scenario (BAU).

Fig. 5 presents the comparison of the six (sub)scenarios to the BAU scenario. The comparison was made through the indicator *Q_i_*(*t*), for *t* equal to 25 years. The first sub-group of bars refers to the comparison of the Co that stays in use (*Q_I_*). It is observed that the main difference is presented in scenario ‘Combined’, where after 25 years Co stays in use around 50% more than in the baseline scenario. Only this scenario and sub-scenarios ‘Collection rate-max10’ and ‘Collection rate-max20’ have an impact of more than 15% (in absolute terms) on the ‘2015 EU-Co in finished products’ that stays in high-end products. The lowest difference is presented in sub-scenario ‘Collection rate-policy20’, which presents around 5% of difference compared to the baseline scenario.

It is important to remember that scenario ‘Combined’ is a combination of the scenarios ‘Collection rate-max5’, ‘Increase production-mobility battery’, and ‘No export’. Of these, the first and the third scenarios are the main contributors to the results of scenario ‘Combined’. A higher collection-to-recycling rate (fewer losses by non-selective collection) and no export of EoL products and recycled material generate that a higher amount of the ‘2015 EU-Co in finished products’ is available to be embedded in new products, reason why Co stays longer in use. It is noteworthy that, separately, these scenarios do not have a significant impact on the Co in use, but that their combined effect on this form of distribution is significant.

The second sub-group of bars shows the comparison of the hoarded Co (*Q_H_*). Clearly, after 25 years the scenarios do not present a significant impact on the amount that stays hoarded. However, this is only true when the complete study period is considered. The main effects on the hoarded Co from the changes taking place in the scenarios occur in the first 10 years of the considered time span.

The third sub-group of bars compares the percentage that is exported (*Q_E_*) after 25 years. Significant differences are observed in sub-scenarios ‘Collection rate-max10’, ‘Collection rate-max20’, and scenarios ‘No export’, and ‘Combined’. The biggest difference is for scenario ‘No export’, followed by sub-scenario ‘Collection rate-max20’. Scenario ‘No export’ considers no export of EoL products and recycled material, therefore it was expected a big difference in this amount compared to the baseline scenario. Indeed, in this scenario the exported Co is almost 50% less than in the BAU scenario. However, ‘Collection rate-max20’ is not directly related to the export. It considers an increase of the collection-to-recycling rate to 100% for all product categories (except for dissipative uses), with a constant increase of 20% per year. In this scenario, the amount exported increases in more than 30% compared to the BAU scenario. The reason is that when increasing the collection-to-recycling rates, the hoarding and post-consumer disposal rates decrease proportionally. This makes more of the ‘2015 EU-Co in finished products’ available for the production of secondary material and final products, contributing to a higher export. Same reasoning applies to sub-scenarios ‘Collection rate-max5’ and ‘Collection rate-max10’, although the increase of the exported amount in these two scenarios occurs in a less extent due to their lower annual increase of the collection-to-recycling rates. Scenario ‘Combined’ is a combination of scenarios with opposite effects on the export, reason why it presents a lower difference than scenario ‘No export’.

Finally, the fourth sub-group of bars presents the difference of the percentage that is physically lost (*Q_L_*). It is observed that the main differences are in sub-scenario ‘Collection rate-max20’ and scenario ‘No export’. Contrary to the previous graph, sub-scenario ‘Collection rate-max20’ decreases the losses and scenario ‘No export’ increases the losses, both in average of around 20%. It is logic that when the export decreases the losses increase and vice versa. A lower export means that more of the ‘2015 EU-Co in finished products’ stays in the region, which will be eventually lost during the studied period due to non-selective collection, the downcycling, and the inefficiencies of the involved processes. However, this result has to be considered carefully. A higher export does not mean necessarily lower physical losses for the global economy, it only causes less Co available in the region. One of the limitations of this model is that it does not allow tracking what happens with the exported Co. Sub-scenario ‘Collection rate-max20’ contributes to have fewer losses not only due to the increase of the export, but also because the non-selective collection rate decreases significantly. The effect of the gradual increase of the collection rates extends the permanence of the ‘2015 EU-Co in finished products’ (as a functional metal in the technosphere) for a couple of years. The rest of the stock is still lost, mainly by downcycling and pre-treatment losses. At the end of the period, the downcycled Co represents around 10% of the initial stock (40% more than in the baseline), and the pre-treatment losses around 13% (60% more than in the baseline). The explanation of higher losses by downcycling and pre-treatment is that higher collection rates mean more secondary Co available for production, both for high-end and low-end products. Due to the distribution of the secondary metal, Co stays longer in the technosphere in the form of hard metals and superalloys, which are the product categories with the lowest pre-treatment efficiencies (together with magnets and other metallic uses).

Regarding indicator π, all scenarios and sub-scenarios present the same half-life time of Co in use than the baseline scenario (6 years).

It is important to mention that indicators could be calculated from the results of the model. Here, two were presented as a way to compare the results and to illustrate the potential of the model. Further research may consider the development of other indicators to assess the circularity of material, and in particular of Co.

## 4 Conclusions

The present research contributes to a better understanding of the societal metabolism of materials. An adapted and expanded version of the model MaTrace was developed, including new features such as the hoarding of end-of-service products and a separate stream for export. The model was applied to forecast the fate of cobalt (Co) initially embedded in ten high-end product (sub)categories, advancing the understanding of Co cycle's in the society. The baseline scenario showed that after 25 years, around 8% of the initial Co (‘2015 EU-Co in finished products’) stays in use, 3% is being hoarded, 28% has been exported, and 61% has been lost. The main losses are due to non-selective collection.

The model depends on a high number of parameters, which makes data a crucial aspect. To assess the reliability of the results, a sensitivity analysis was performed for 56 of the 242 parameters of the model. These 56 parameters considered parameters with multiple values or with a single assumed value. Thirteen of them showed an effect of more than 15% (in absolute terms) on the distribution of Co. Some of the parameters had a higher impact than others, depending on how different their minimum and maximum values were from each other. From the results, it is clear that robust and sound values are required in order to have more reliable results. Important is to indicate that this analysis did not target to assess the sensitivity of the model, but of this particular case study.

Nine other scenarios were built, introducing changes in the collection-to-recycling rate, the production of mobility batteries, and the export of EoL products and recycled material. It was observed that the highest impacts come from the scenarios where the collection-to-recycling rates increase gradually to 100%, and/or where there is no export. In these, the amount of ‘2015 EU-Co in finished products’ that stays in use increases between 20 and 50%. A clear outcome of the analysis of these scenarios is that in order to extend the time that Co stays in use in high-end products in the EU, higher recycling-to-collection rates are required, together with a lower export of the material.

Despite the potential of the model, attention should be paid to some of its limitations: (1) it does not capture the Co that enters to the economy after 2015, (2) it does not track the fate of the exported Co, which represents after 25 years, between 15 and 38% of the initial stock of Co (depending on the scenario). However, despite these limitations, the model allows identifying ‘hotspots’ and quantifying losses; identifying when Co becomes available; and identifying where there is room for improvement. Furthermore, the results of the model can be used as a basis for different circularity indicators, such as the ones presented here.

From the obtained conclusions, some recommendations can be given to policy makers. As it was indicated, the main losses come from the non-selective collection. Hence, to make Co staying longer in the EU's economy, better take-back systems should be established. The focus should be on portable batteries and hard metals, which are two of the main applications of Co that in addition present the highest post-consumer disposal rate. Another aspect that can be steered through policy is the amount of Co exported from the EU. Co could stay longer in the EU if more secondary resources are treated and used domestically. Finally, in order to obtain more reliable results, policy makers could also encourage industries to make information more transparent and available, for example, regarding processing, manufacturing, and recycling yields per material.

Further research may consider to include what enters to the economy after the initial year, adding the production and import of material and final products along the study period. To do so, it will be necessary to predict the domestic extraction of primary Co; together with the imports of primary Co, intermediates, processed material, final products, scrap, and EoL products. The prediction could be done looking at the historical data of the trade of Co and associated materials and products, using data sources such as Eurostat or UN Comtrade. In addition, other scenarios could be studied in order to analyze the effect of changes in technology and/or public policies. Finally, the model could be applied to study other (critical) materials, to better understand their metabolism; and to study energy consumption related to the production and use of secondary material.

## Declaration of Competing Interest

The authors declare that they have no known competing financial interests or personal relationships that could have appeared to influence the work reported in this paper.

## Appendix Supplementary materials

Image, application 1

Image, application 2
