# Supplementary material for: Cobalt in end-of-life products in the EU, where does it end up? - The MaTrace approach
Source: Resour Conserv Recycl. 2020 Jul;158:104842. doi: 10.1016/j.resconrec.2020.104842 (PMC7185230; doi:10.1016/j.resconrec.2020.104842)
Supplement: Supplementary file 1 [file mmc1.docx]

**Supplementary Material**

*for*

**Refining the Understanding of China's Tungsten Dominance with Dynamic Material Cycle Analysis**

Linbin Tang ^1,2,3^, Peng Wang ^1,2 *^, T.E. Graedel ^4^, Stefan Pauliuk ^5^, Keying Xiang ^1,2^, Yan Ren^1,2,*^and Wei-Qiang Chen^1,2,3^

^1^ Key Lab of Urban Environment and Health, Institute of Urban Environment, Chinese Academy of Sciences, 1799 Jimei Road, Xiamen, Fujian 361021, China

^2^ Fujian Innovation Academy, Chinese Academy of Sciences, 1799 Jimei Road, Xiamen, Fujian 361021, China

^3^ University of Chinese Academy of Sciences, No.19(A) Yuquan Road, Beijing 100049, China

^4^ Center for Industrial Ecology, School of Forestry & Environmental Studies, Yale University, New Haven, CT 06511, USA

^5^ Industrial Ecology Group, Faculty of Environment and Natural Resources, University of Freiburg, Tennenbacher Strasse 4, D-79106 Freiburg, Germany

* Corresponding authors:

Peng Wang, [pwang@iue.ac.cn](mailto:pwang@iue.ac.cn)

Yan Ren, [yanren@iue.ac.cn](mailto:yanren@iue.ac.cn)

**Table of Contents**

[S1 Tungsten Cycle Construction and Quantification 1](#_Toc35165008)

[S1.1 System quantification 1](#_Toc35165009)

[S1.2 System Definition 4](#_Toc35165010)

[S1.3 Parameters and data sources 8](#_Toc35165011)

[S2 Results 13](#_Toc35165012)

[S2.1 Overview 13](#_Toc35165013)

[S2.2 Details of China’s tungsten stocks and flows 17](#_Toc35165014)

[S2.3 Mass balance 19](#_Toc35165015)

[References 21](#_Toc35165016)

## S1 Tungsten Cycle Construction and Quantification

### S1.1 System quantification

**Fig. S1** represents the framework of the material flow quantification as used in this paper, and the following describes the detailed quantification process and parameters for material flow and stocks at each life stage. The principle of mass balance is applied in the quantification process.

**Fig. S1 Material flow quantification framework** *(M&B= Mining and Beneficiation stage; F&M= Fabrication and Manufacturing stage; EU= End use stage; WM= Waste Management; L= Loss)*

**Table S1 Quantification process and parameters for the framework**

| **Symbol** | **Explanation** | **Equations** | **Stage** |
| --- | --- | --- | --- |
| $C_{w}$ | Tungsten content in tungsten-containing products | Based on the technical report and existing literature |  |
| ${FR}_{i}$ | Fabrication rate in the production process of $i$ products | Based on the technical report and existing literature |  |
| ${LR}_{i}$ | Loss rate in the production process of $i$ products | ${LR}_{i}=1-{RR}_{i}$ |  |
| $Q_{i}$ | Amount of tungsten-containingg material and products in stage $i$ | Based on Statistics data |  |
| ${MS}_{n}$ | Market share of end-use sector *n* |  |  |
| $T_{i, t}$ | Obsolete share of end-use sector $i$ after serving *t* in EU stage | probability density of the lifetime distribution function |  |
| ${RU}_{i}$ | Amount of recycling use from the WM stage | Mass balance |  |
| (1.a) | Input for M&B stage | $\left( 1.a \right)=(1.b)/{FR}_{M\&B}$ | Mining  &  Beneficiation  (M&B) |
| (1.c) | Trade flows for tungsten products in stage 1. | ${(1.c)}^{import}=Q_{M\&B}^{import}\times C_{w}$  ${(1.c)}^{export}=Q_{M\&B}^{export}\times C_{w}$ |  |
| (1.d) | Loss in M&B stage | $(1.d)=(1.a)-(1.b)$ |  |
| (2.a) | Input for F&M stage | $\left( 2.a \right)=\left( 1.b \right)$ | Fabrication  &  Manufacturing  (F&M) |
| (2.b) | Output for F&M stage | $\left( 2.b \right)=\left( 2.a \right)\times{FR}_{F\&M}$  Or $\left( 2.b \right)=Q_{F\&M}^{production}\times C_{w}$ |  |
| (2.d) | Loss in F&M stage | $(2.d)=(2.a)-(2.b)$ |  |
| (2.c) | Trade flows for tungsten products in the F&M stage. | ${(2.c)}^{import}=Q_{F\&M}^{import}\times C_{w}$  ${(2.c)}^{export}=Q_{F\&M}^{export}\times C_{w}$ |  |
| (3.a) | Input for EU stage | $\left( 3.a \right)=\left( 2.b \right)$ | End Use  (EU) |
| (3.b) | Output from EU stage | $\left( 3.b \right)=\left( 3.a \right)\times T_{i,t}$ |  |
| IU | In-use stock | $\Delta IU=(3.a)-(3.b)$ |  |
| (3.d) | Dissipate loss from EU stage | $\left( 3.d \right)=\left( 3.a \right)-\left( 3.b \right)-\Delta IU$ |  |
| (4.c) | Trade flows for tungsten products in WM stage. | ${(4.c)}^{import}=Q_{\mathrm{WM}}^{import}\times C_{w}$  ${(4.c)}^{export}=Q_{WM}^{export}\times C_{w}$ | Waste Management  (WM) |
| (4.d) | Loss from WM stage | $\left( 4.d \right)=\left( 4.a \right)-{RU}_{i}$ |  |

### S1.2 System Definition

**Fig. S2** represents the system boundary for our dynamic material flow analysis of tungsten. The following section described detailed of the tungsten life cycle in each stage. Notably, all stocks and flows in the framework are converted into the metal content for consistency.


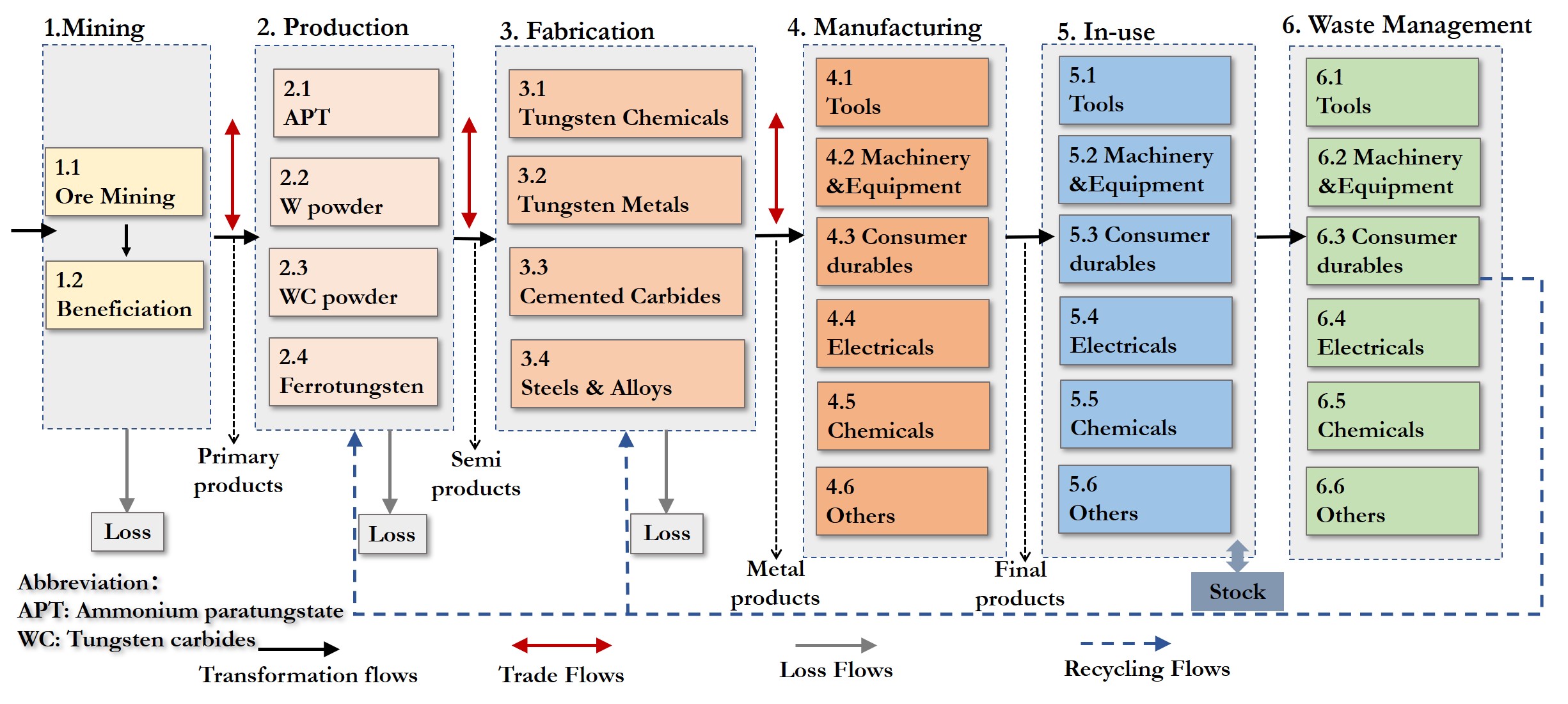


**Fig. S2 Framework for tungsten stock and flow analysis** *(The Mining stage is where the tungsten ores are mined and beneficiated to tungsten concentrates; Production, fabrication, and manufacturing stage are comprised of the transformation from the tungsten concentrates through semi products and metal products to final products. The in-use stage is where final products are employed. Afterward, the products are discarded into the waste management and recycling system)*

Tungsten's name originates from a Swedish word “Tungsten” which means “heavy stone”. When it’s found in nature, this metal has a silvery – gray color, and it is resistant to acid, bases, and heat. This metal is extremely durable, which indicates it can uphold a lot of pressure. Tungsten has the highest melting point among all metals; this is one of the reasons why it’s presented in manufacturing industries, especially in heavy industry.

We establish a framework to tracing the tungsten flows from tungsten mining and concentrate stage through fabrication, manufacturing, end use, into waste management stage(Brunner and Rechberger, 2003; Harper and Graedel, 2008). The detailed explanations are given as follows:

**S1.2.1 Mining**

More than 20 species of tungsten-bearing minerals are known, but only minerals in the wolframite group and scheelite are important ores sources. All members of the wolframite series are called “black ores” and occupies the half of the world’s tungsten reserves. Scheelite is a calcium tungstate with major economic importance, accounting for about 50% of the world’s deposits.

Mining and beneficiation are referred to as the production stage. Most tungsten is mined from underground mines. A few tungsten mines have open-pit methods to access near-surface ores. Tungsten ores are crushed, ground, and concentrated by a combination of gravity separation, flotation, or magnetic methods (Stafford, 1988), then produce a concentrate containing 50%-70%WO_3_. We defined as the standard metric ton, which tungsten concentrates with 65% WO_3_/ per metric ton. Tungsten recovery by these concentration methods ranges about 75% (Leal-Ayala et al., 2015).

**S1.2.2 Production**

In this stage, there are four types of semi products are produced:

**(1) Ammonium para-tungstate (APT).** About a half of tungsten ore concentrates are processed chemically to produce ammonium para-tungstate (APT), which is the main tungsten semi-mineral product traded in the market. and the most essential raw material to further processing other products, such as tungsten metal powder, tungsten chemicals.

**(2) Tungsten metal powder.** Tungsten powder is produced from AMT or tungstic oxide through the hydrogen-reduced method, which is a primary consideration in its use for manufacturing tungsten mill products or tungsten carbides products.

**(3) Tungsten carbides powders.** When tungsten metal powder is carburized, compacted with cobalt binder into the desired shape, and sintered, the tungsten carbide powders is accomplished. The carbide powders usually have three types: conventional, crystalline, and cast tungsten carbides. The conventional one is the most essential application of tungsten carbide powder, accounting for more than 90% of tungsten carbide use. Therefore, we assume that the production of tungsten carbide powder is all conventional tungsten carbide.

**(4) Ferrotungsten**. Tungsten ore concentrates can also be used to make ferrotungsten, which is a master alloy used in the production of steel. It can be made using either carbothermic or carbothermic-silicothermic reduction. Tungsten scrap also can be used as raw materials to make ferrotungsten.

**S1.2.3 Fabrication**

In this stage, there are four types of metal products are produced:

**(1) Tungsten chemicals.** Tungsten blue oxide, tungstic acid, tungsten oxide, tungsten trioxide, tungstic acid, and ammonium meta-tungstate are called tungsten chemicals. APT is the raw material to produce tungsten chemicals. APT is calcined under reducing conditions to form tungsten blue oxide, which is a mixture of tungsten oxides and other constituents; calcined under oxidizing conditions to form tungsten trioxide; decomposed by hydrochloric acid to form tungstic acid; or subjected to partial thermal decomposition to form ammonium meta-tungstate (AMT) (Christian et al., 2011).

**(2) Tungsten mill products.** Tungsten metal powder is compressed, sintered, heated in incipient fusion by the passage of electric current, swaged, and drawn or rolled into tungsten mill product forms, such as wire, rod, and sheets (Mitchell, 2010).

**(3) Cemented carbides.** These are also referred to as “hard metal”. are sintered powder metallurgical parts that are used as cutting tools and wear-resistant components by the construction, metalworking, mining, and oil drilling industries. These are made from tungsten carbides powder (Schubert et al., 2010).

**(4) Steels and alloys.** Ferrotungsten can be used as an alloying additive for tungsten in steels and alloys making and casting. It is used in the refining of high-speed steel, tool steel, and superalloys (Jones et al., 2017; Gunn, 2014).

**S1.2.4 Manufacturing**

In this stage, metal products are used to make final products for six end-use sectors: Tools, Machinery & Equipment, Consumer durables, Electricals, Chemicals, and Others (e.g., cutting tools, electrode, lighting, mobile phone vibrator, pigment, and catalysis, etc.). We referenced the end-use sectors categories delineated by the International Tungsten Industry Association (ITIA) (Moll, 2016; ITIA, 2018). Due to the limitation of data, we integrated the eight in-use sectors into six according to the tungsten metal product attributes.

**S1.2.5 In use**

In this stage, Tungsten enters the consumer market in the form of various final tungsten-containing products. Because the final products which embodied tungsten are so miscellaneous and the tungsten’s content is always low that it is difficult to identify accurately, the tungsten flows thus become highly sophisticated and statistics are sparse. We classify final tungsten products into six sectors, and the rules of classification are as follows in Fig. S2.

**S1.2.6 Waste management**

Tungsten may be lost from final products in essentially three ways. These include dissipation, discard, and dilution. The discarded final products which reach their end-of-life are processed in the waste management stage. The stage is concluded in three sub-stages: collection and separation, incineration, and landfill. Due to the lack of statistical data regarding the recycling rate of end-of-life tungsten products in China, we estimated such value based on the end-of-life product waste (in tungsten content) generation and the demand of recycled tungsten products as resource for production. To be more specific, we quantified the input and output of the production and fabrication stage, identified the gap where the input from the primary resource is less than the output, and inferred this gap as the output (i.e. recycled end-of-life scrap) from the recycling stage according to the mass balance principle.

### S1.3 Parameters and data sources

**Table S2 Summary of parameters and data sources**

| **Data series** | **Sources** | **References** |
| --- | --- | --- |
| **Mining** | | |
| Tungsten ores grades | Statistics data from the China Nonferrous Metals Industry Association (CNMIA) | (CNMIA, 1949-2017.) |
| Primary product production in China | Statistics data from the China Nonferrous Metals Industry Association (CNMIA) | (CNMIA, 1949-2017.) |
| Tungsten concentrates |  |  |
| Yield | Statistics data | (CNMIA, 1949-2017.) |
| Content | 51% | Calculated by atomic weight |
| Recovery rate | 75% | (Leal-Ayala et al., 2015) |
| HS code | 261100 | (United Nations, 2015) |
| Price | 12650 ($/ton, 9/30/2019) | (CBC Website) |
| **Production** | | |
| APT |  |  |
| Yield | Statistics data from 1985 to 2017  Calculated by market share before 1985 | (CTIA, 1985-2017) |
| Market share | 50% in 1985 assumed keep constant before | (CTIA, 1985-2017) |
| Content | 70% | (Leal-Ayala et al., 2015) |
| Fabrication rate | 96% | (Leal-Ayala et al., 2015) |
| HS code | 284180 | (United Nations, 2015) |
| Price | 19537 ($/ton, 9/30/2019) | (CBC Website) |
| W powder |  |  |
| Yield | Statistics data from 1985 to 2017  Calculated by market share before 1985 | (CTIA, 1985-2017) |
| Market share | 80% in 1985 assumed keep constant before | (CTIA, 1985-2017) |
| Content | 97% | (Leal-Ayala et al., 2015) |
| Fabrication rate | 74% | Back Calculation |
| HS code | 810110 | (United Nations, 2015) |
| Price | 29411 ($/ton, 9/30/2019) | (CBC Website) |
| W carbide powder |  |  |
| Yield | Statistics data from 2000 to 2017  Calculated by market share before 2000 | (CTIA, 1985-2017) |
| Market share | 10% from 1949 to 1960, 30% from 1961 to 1970, 50% from 1971 to 1980, and 70% from 1981 to 2000 | (CTIA, 1985-2017) |
| Content | 94% | Calculated by atomic weight |
| Fabrication rate | 99% | (Leal-Ayala et al., 2015) |
| HS code | 284990 | (United Nations, 2015) |
| Price | 28991 ($/ton, 9/30/2019) | (CBC Website) |
| Ferrotungsten |  |  |
| Yield | Statistics data from 1985 to 2017  Calculated by market share from 1949 to 1984 | (CTIA, 1985-2017) |
| Market share | 50% in 1985 assumed keep constant before | (CTIA, 1985-2017) |
| Content | 75% | Calculated by atomic weight |
| Fabrication rate | 68% | (CTIA, 1985-2017) |
| HS code | 720280 | (United Nations, 2015) |
| Price | 20238 ($/ton, 9/30/2019) | (CBC Website) |
| **Fabrication** | | |
| Tungsten chemicals | Sodium tungstate, Ammonium metal tungstate, tungstate acid, and other tungstates are containing |  |
| Yield | Statistics data from 2008 to 2017  Calculated by market share from 1949 to 2008 | (CTIA, 2017) |
| Market share | 6% of the total tungsten consumption average in these years assumed keep constant before | (Zeiler et al., 2018) |
| Content | 70% | (Graedel et al., 2015) |
| Fabrication rate | 93% | (USGS, 2005) |
| HS code | 284180 | (United Nations, 2015) |
| Price | 14775 ($/ton, 9/30/2019) | (CBC Website) |
| Tungsten mill products | Wire, sheets, rods, etc. |  |
| Yield | Statistics data from 2000 to 2017  Calculated by market share from 1949 to 1999 | (CTIA, 2017) |
| Market share | 90% from 1949 to 1960, 70% from 1961 to 1970, 50% from 1971 to 1980, and 30% from 1981 to 2000 | Expert interview (Zhu，Xiusheng, 2018) |
| Content | 99.4% | (Graedel et al., 2015) |
| Fabrication rate | 95% | (USGS, 2005) |
| HS code | 810192/8101950/810191/810194/810193/810196 | (United Nations, 2015) |
| Price | 37815 ($/ton, 9/30/2019) | (CBC Website) |
| Cemented Carbides | Cutting tools, wear-resistant parts, milling alloy, and others |  |
| Yield | Statistics data from 1949 to 2017 | (CNMIA, 1949-2017) |
| Content | 85% | (USGS, 2005) |
| Fabrication rate | 97.5% | (Graedel et al., 2015) |
| HS code | 820900 | (United Nations, 2015) |
| Steels & alloys | Heavy metal alloys, high-speed steel, superalloys, tool sheets, etc. |  |
| Yield | Statistics data from 1983 to 2017  Calculated by the production of ferrotungsten from 1949 to 1982 | (CTIA, 2017; Li, 2005) |
| Content | 7% | (USGS, 2005) |
| Fabrication rate | 100% | Expert interview (Zhu，Xiusheng, 2018) |
| HS code | 722540/722599/722620/722710/722810/722990 | (United Nations, 2015) |
| Price | 10000 ($/ton, 9/30/2019) | (CBC Website) |
| **Manufacturing & In use** | | |
| Tools | Cutting tool inserts, Blades, Broaches, reamers, drawing or extruding dies, milling/turning cutters dies, drill, threading taps |  |
| Yield | Direct calculation |  |
| Market share | 100% of cutting tools in cemented carbide and steels & alloys | (Zeiler et al., 2018) |
| Lifetime | One year in the normal distribution | (Zeiler et al., 2018) |
| Machinery & Equipment | Rock drilling machine, Oil and natural gas drilling machinery, Lathes for removing metal, Machine-tools |  |
| Yield | Direct calculation |  |
| Market share | 100% of wear-resistance parts and milling alloy in cemented carbides are assumed used in Machinery & Equipment | (Gunn, 2014; ITIA, 2018) |
| Lifetime | 10 years in the normal distribution | (Harper, 2008; Harper and Graedel, 2008) |
| Consumer durables | trucks, turbo engine, mobile phone, weight stack, etc. |  |
| Yield | Direct calculation |  |
| Market share | 80% of tungsten mill production | Expert interview (Zhu，Xiusheng, 2018) |
| Lifetime | 10 years in the normal distribution | (Harper and Graedel, 2008) |
| Electricals | electrodes, contact terminal, etc |  |
| Yield | Direct calculation |  |
| Market share | 20% of tungsten mill products | Expert interview (Zhu，Xiusheng, 2018) |
| Lifetime | 1 year in the normal distribution | (Harper and Graedel, 2008) |
| Chemicals | Catalysts, pigment, dyestuff, etc. |  |
| Yield | Direct calculation |  |
| Market share | 100% of tungsten chemicals |  |
| Lifetime | 3 years in the normal distribution | (Graedel et al., 2015) |
| Others | Heavy tank, armor-piercing projectile, etc. |  |
| Yield | Allocation and direct calculation |  |
| Market share | 100% of others in cemented carbides | Expert interview (Zhu，Xiusheng, 2018) |
| Lifetime | 1 year in the normal distribution | (Harper and Graedel, 2008) |

## S2 Results

### S2.1 Overview

Fig. S3 shows an overview of China’s tungsten stocks and flows in specific eight years for revealing the time-evolution characteristics of China’s tungsten.

| 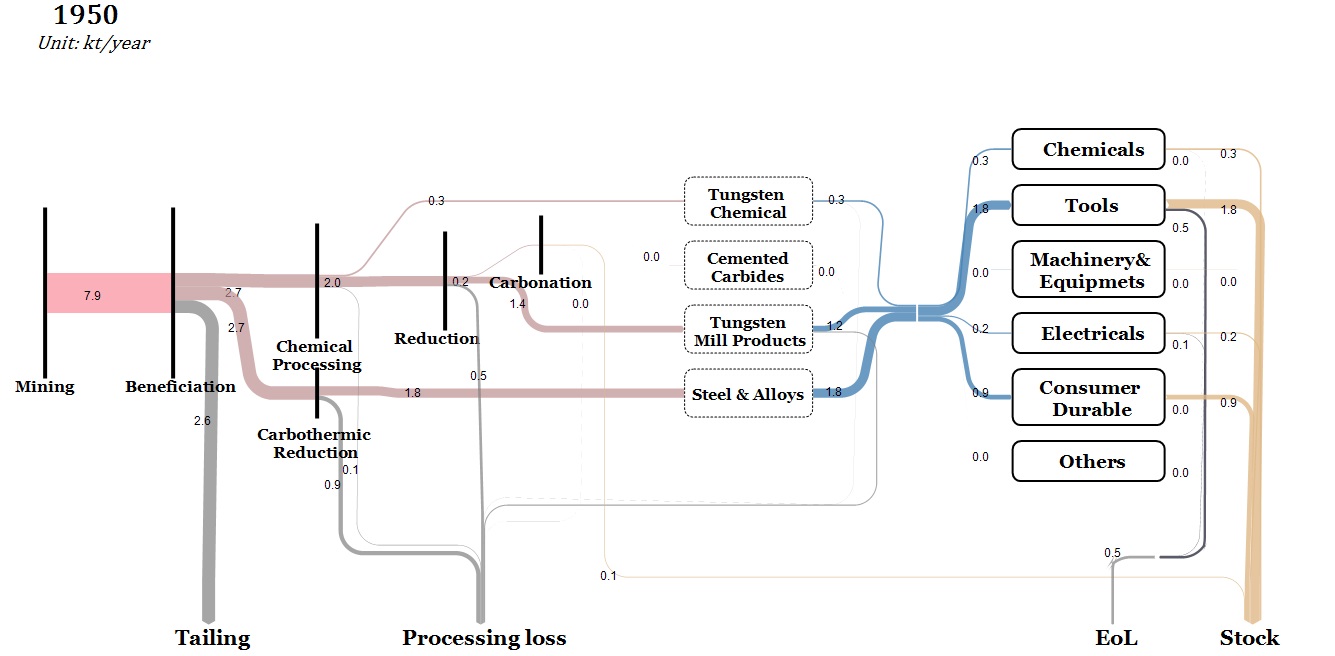 |
| --- |
| 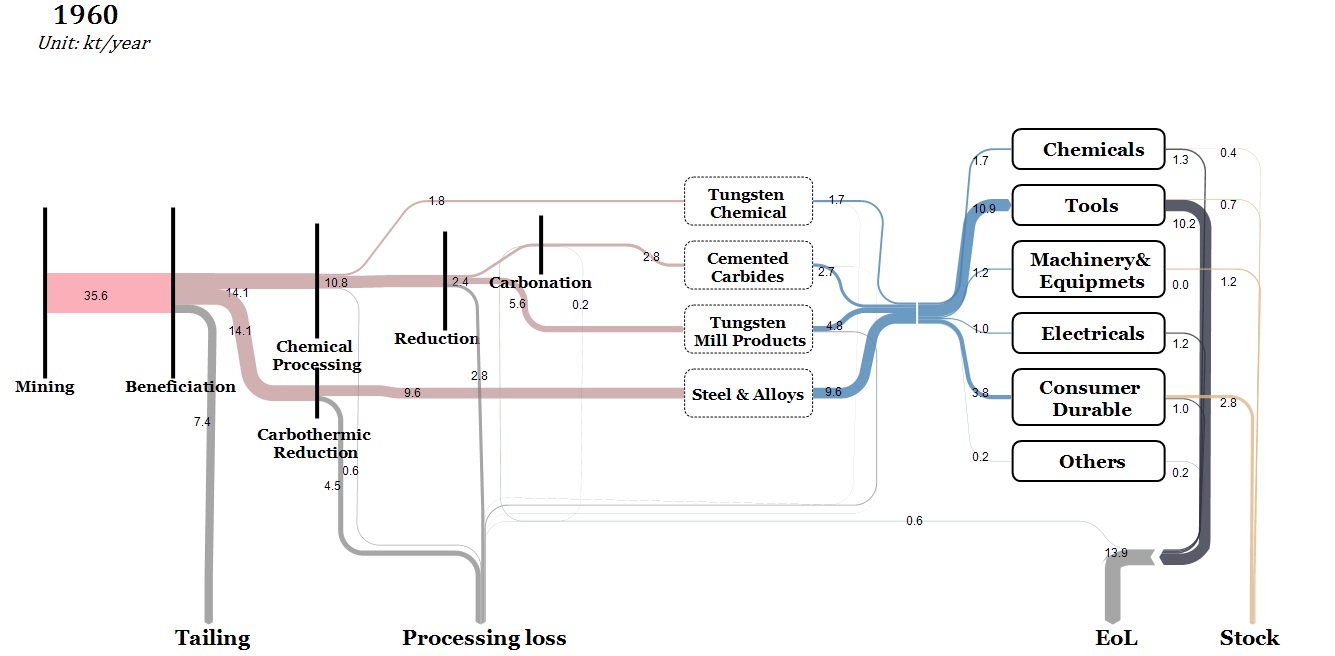 |
| 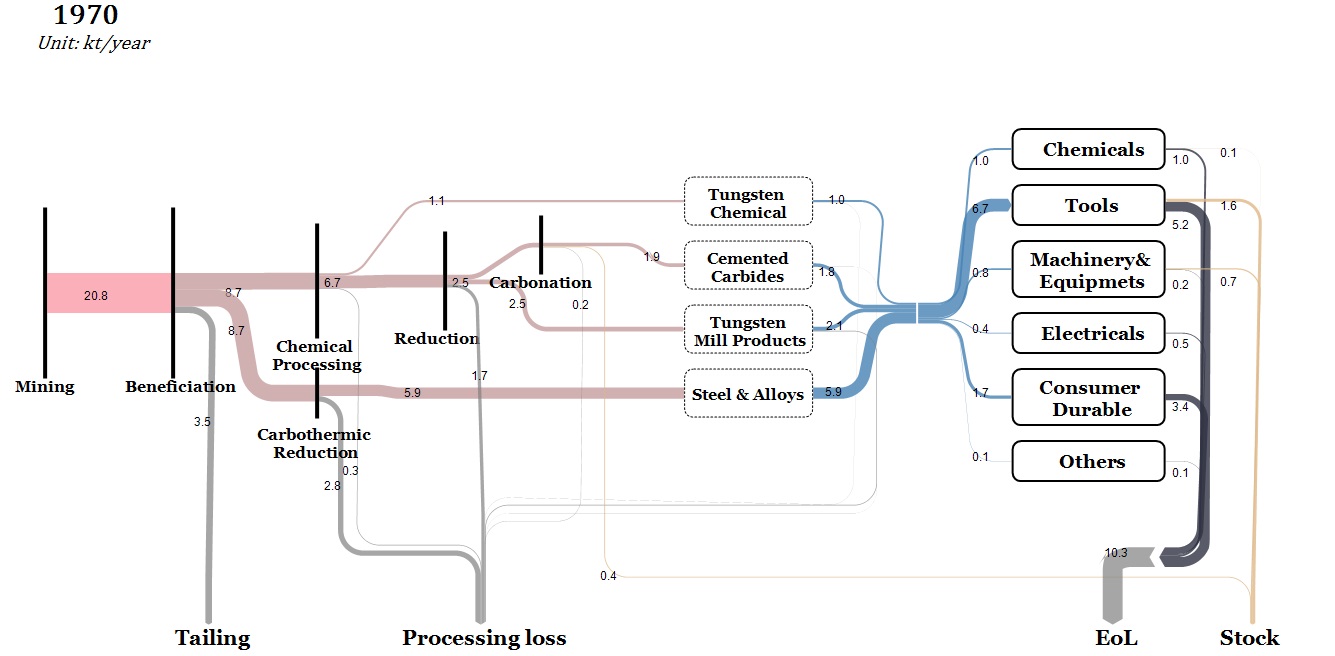 |
|  |
| 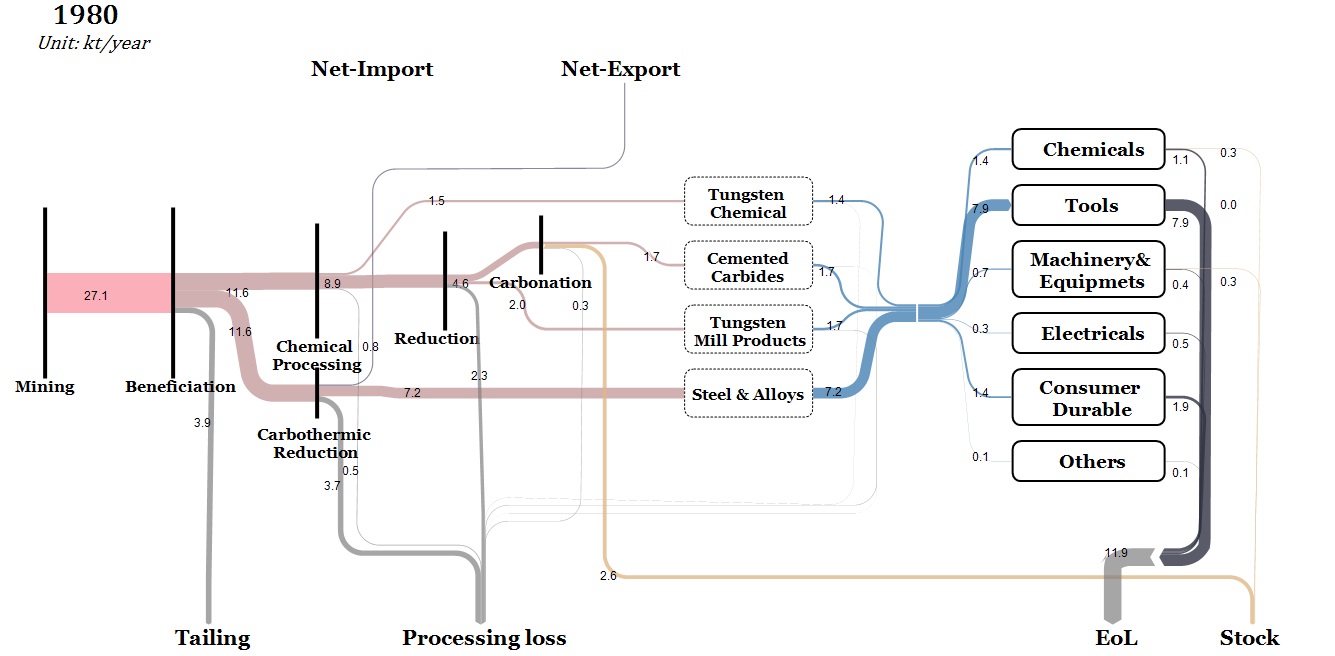 |
|  |
| 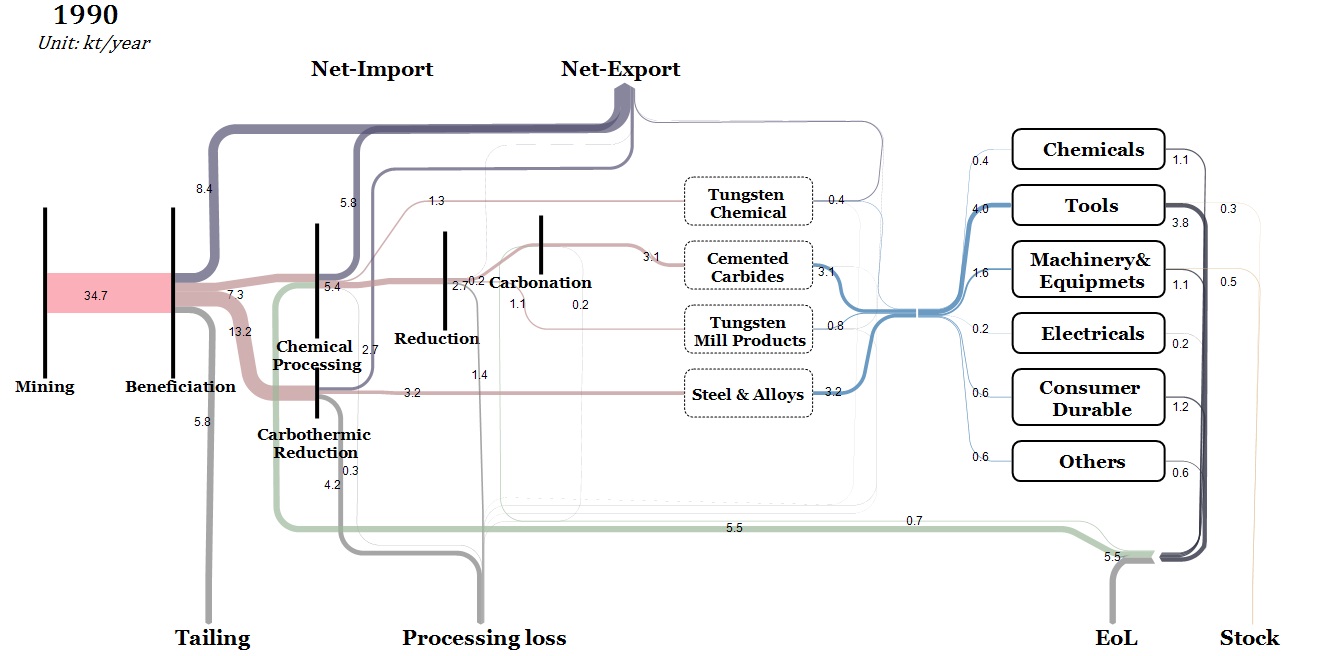 |
| 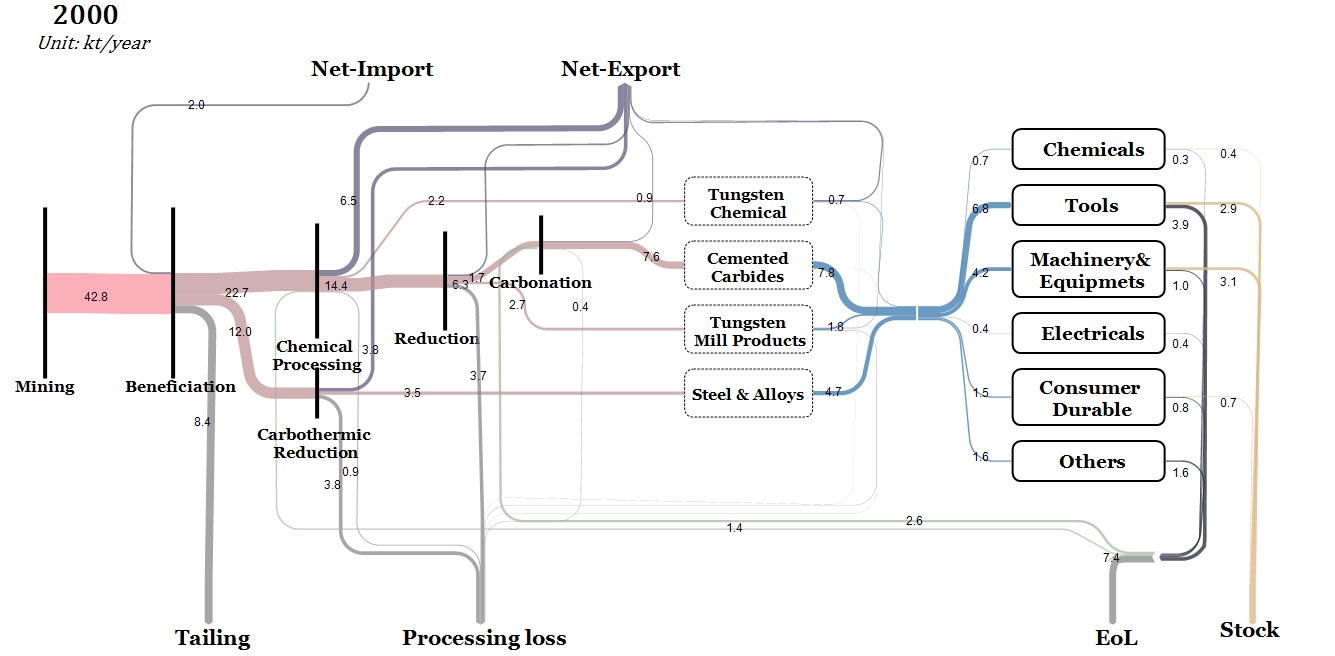 |
|  |
| 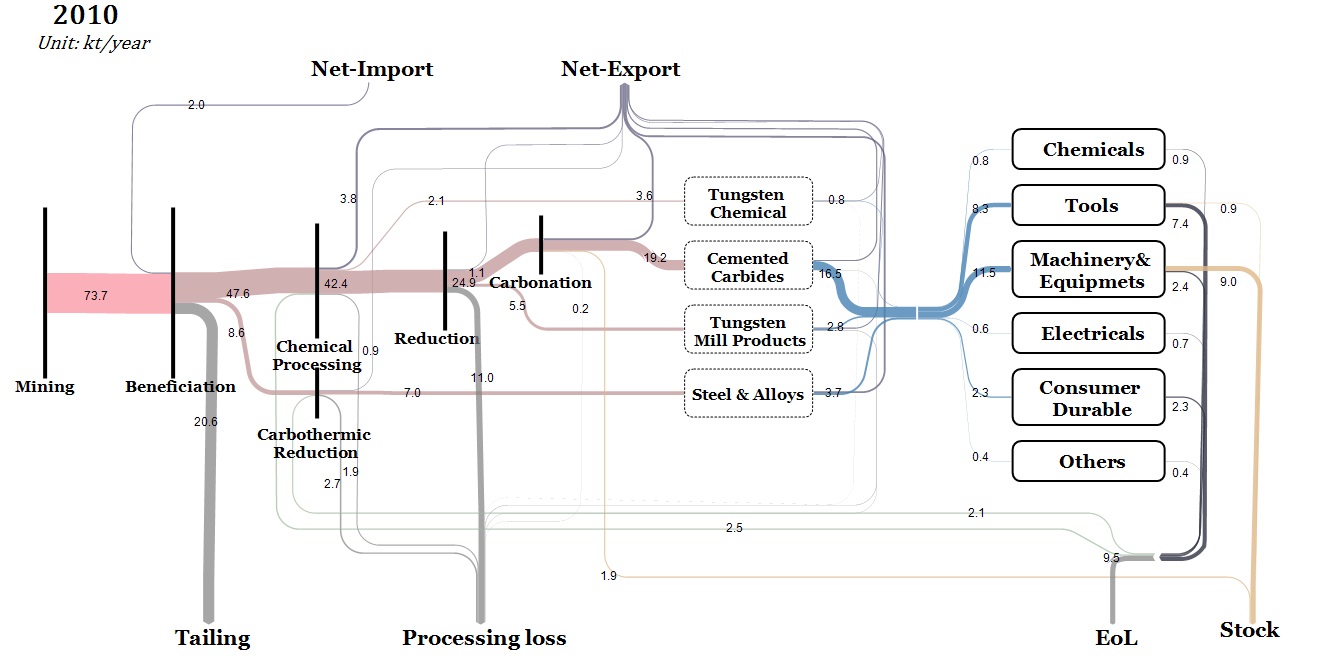 |
|  |
| 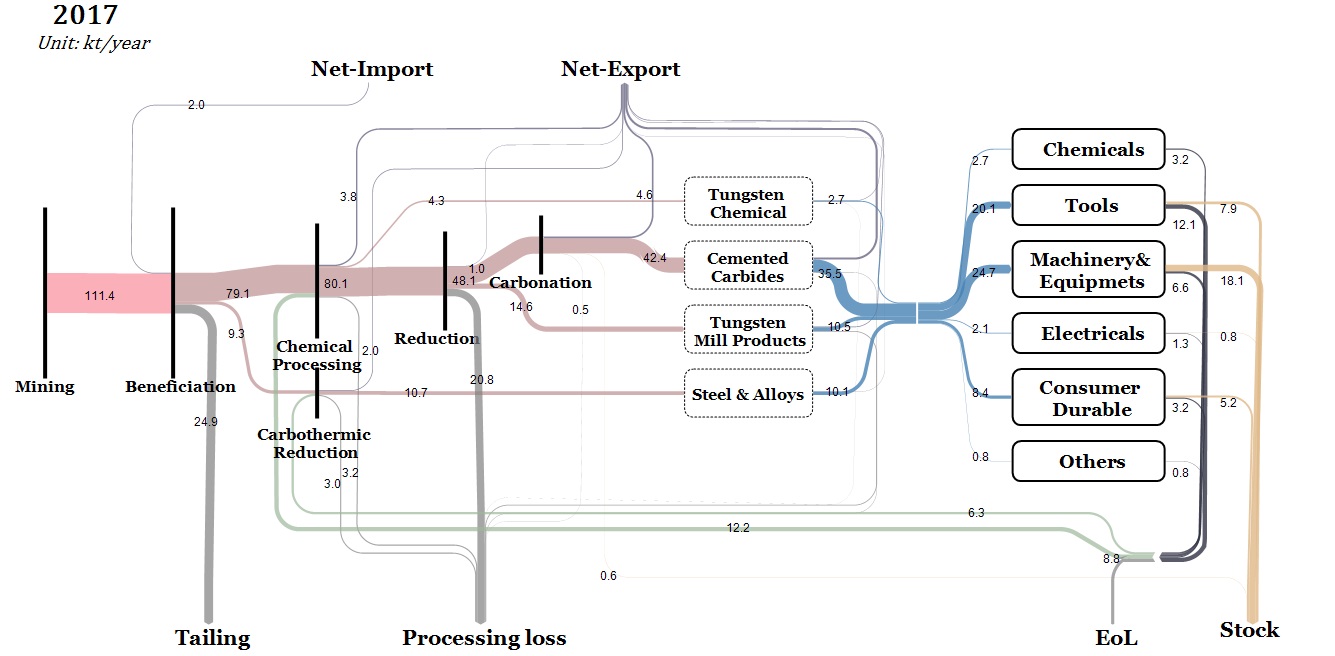 |

**Fig. S3 Sankey diagram of China’s tungsten stocks and flows in specific years** *(All flows present the tungsten use in China in specific years, where the width of the edge represents the weight of each material flow; and the color of flows means the different stages in tungsten’s life cycle)*

### S2.2 Details of China’s tungsten stocks and flows

Fig. S4 represents the detailed information of China’s tungsten stocks and flows from 1949 to 2017.

| 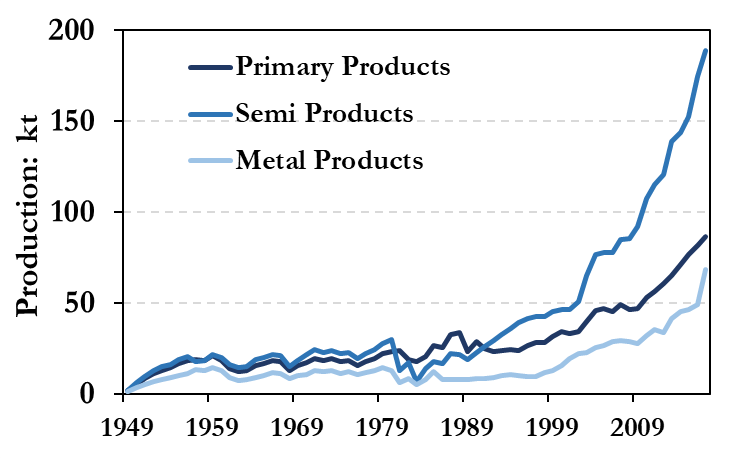 | 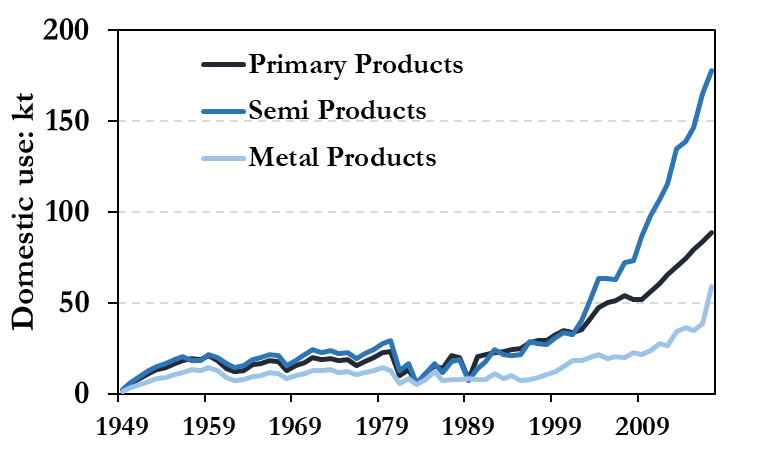 |
| --- | --- |
| a. Annual production | b. Annual domestic consumption |
| 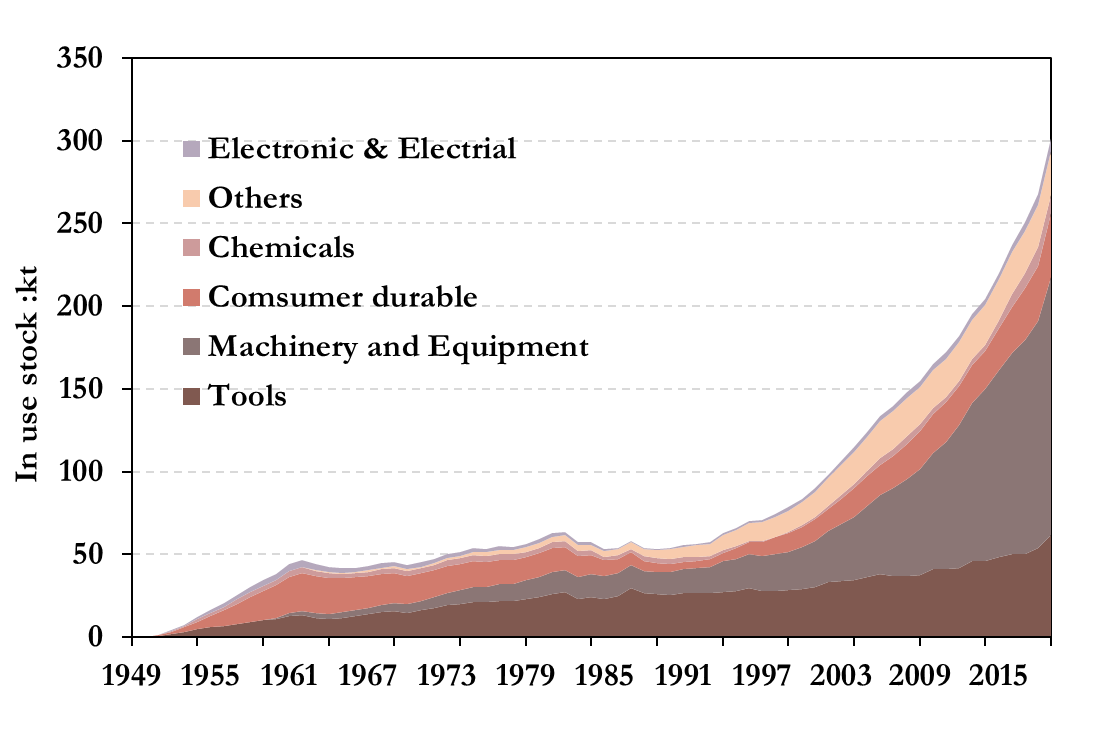 | 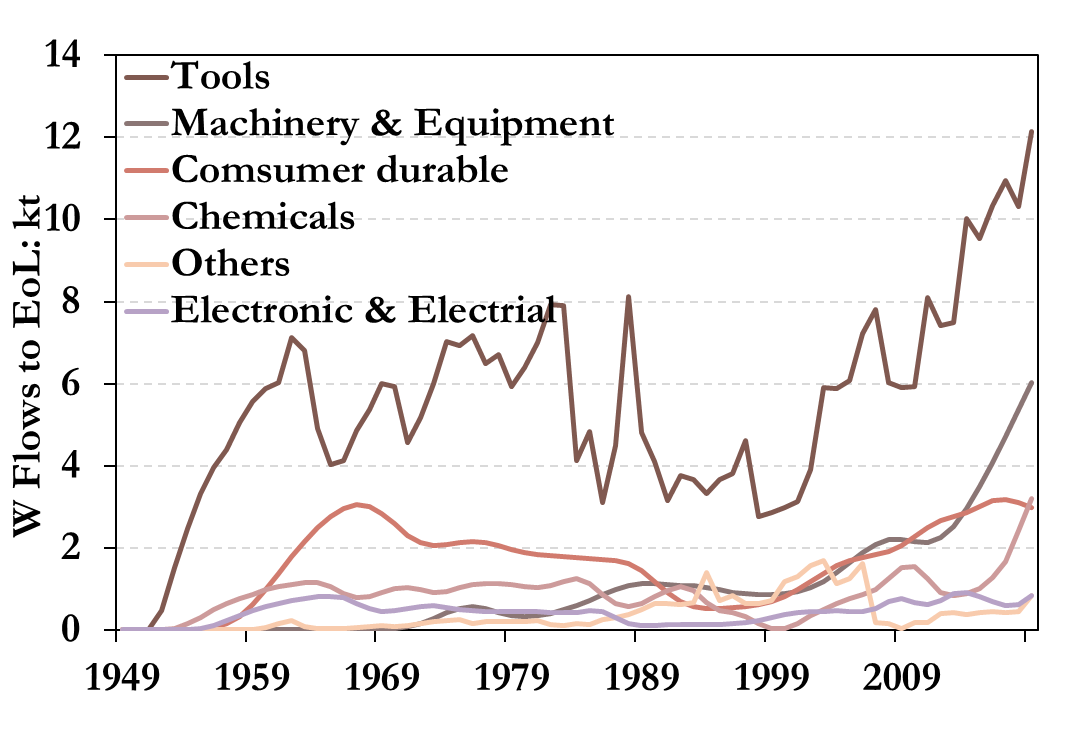 |
| c. In-use stock by sectors | d. Outflows from in-use stock to EoL |
| 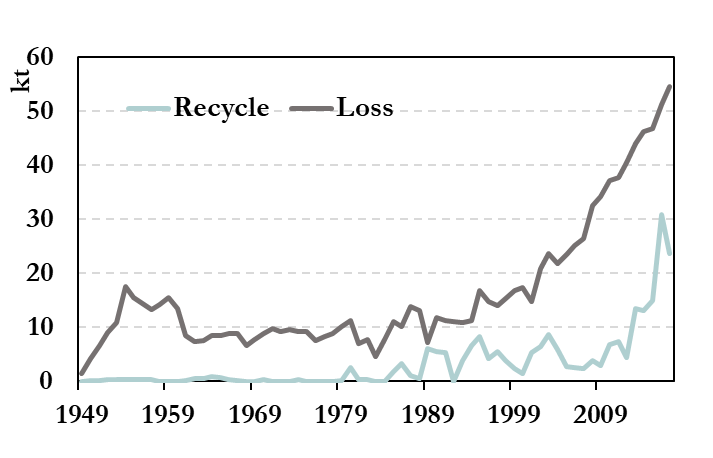 | 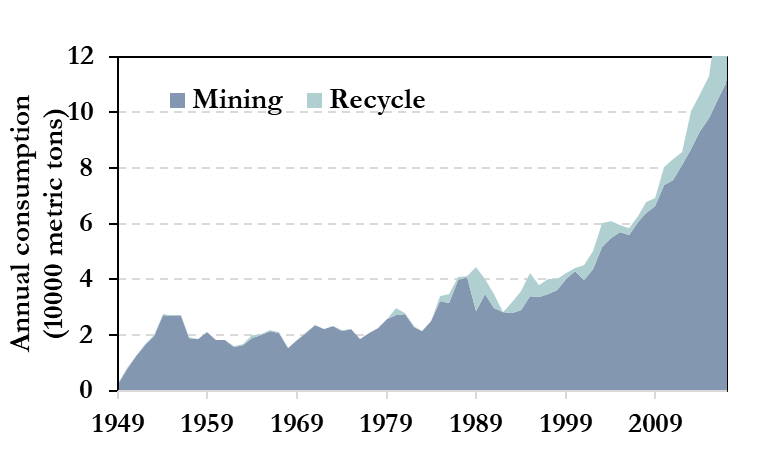 |
| e. Recycle use VS Old scrap loss | f. Recycling VS Mining |
| 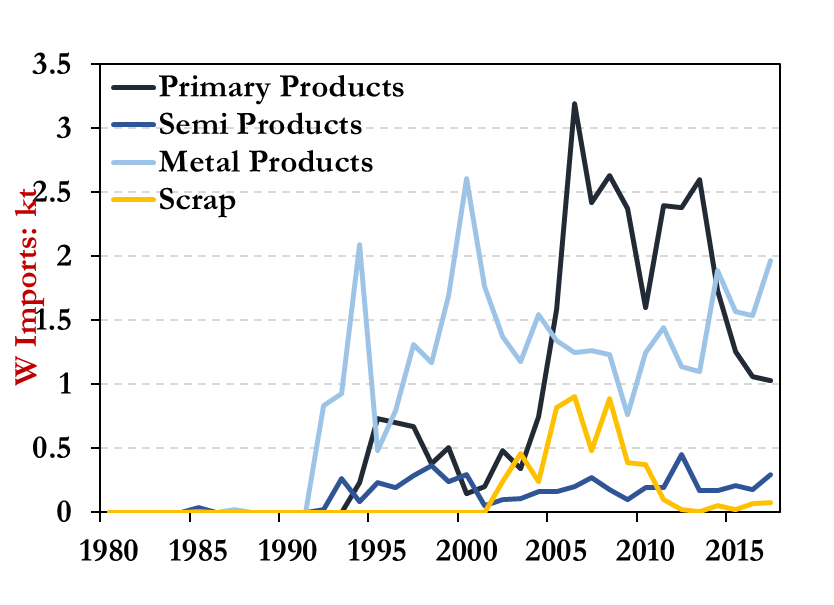 | 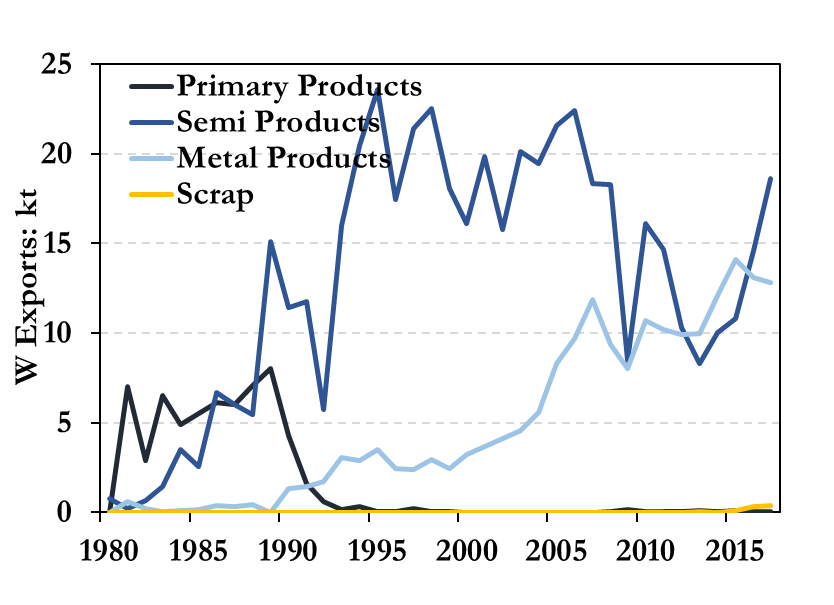 |
| g. Import of China’s tungsten | h. Export of China’s tungsten |
| 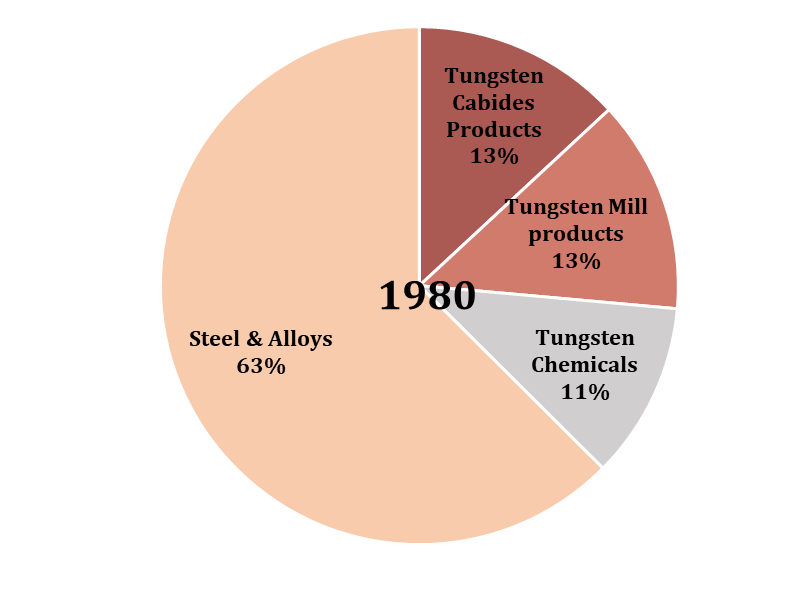 | 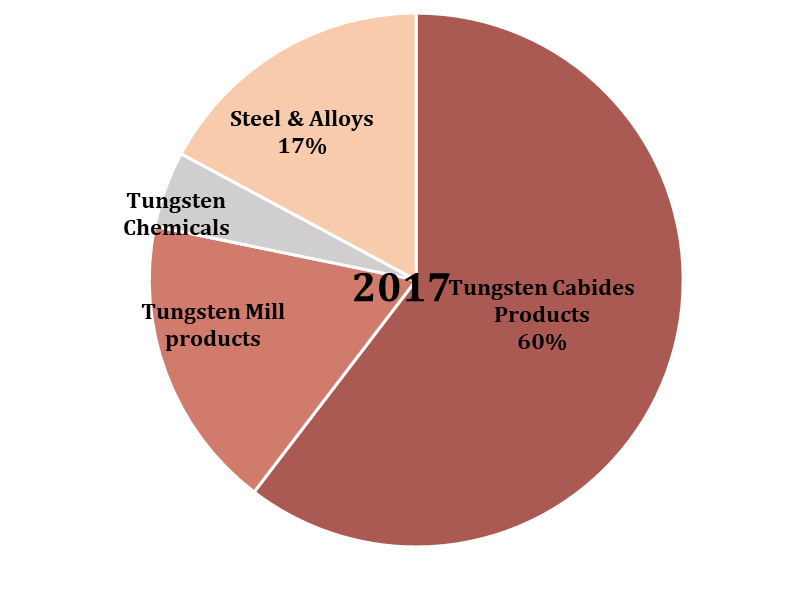 |
| i. Composition of Final-metal tungsten use in 1980 | j. Composition of Final-metal tungsten use in 2017 |

**Fig. S4 China’s tungsten stocks and flows from 1949 to 2017**

### S2.3 Mass balance

**Fig. S6** and **Fig. S5** represents the annual balance between total inputs and total outputs of tungsten in China from 1949 to 2017.


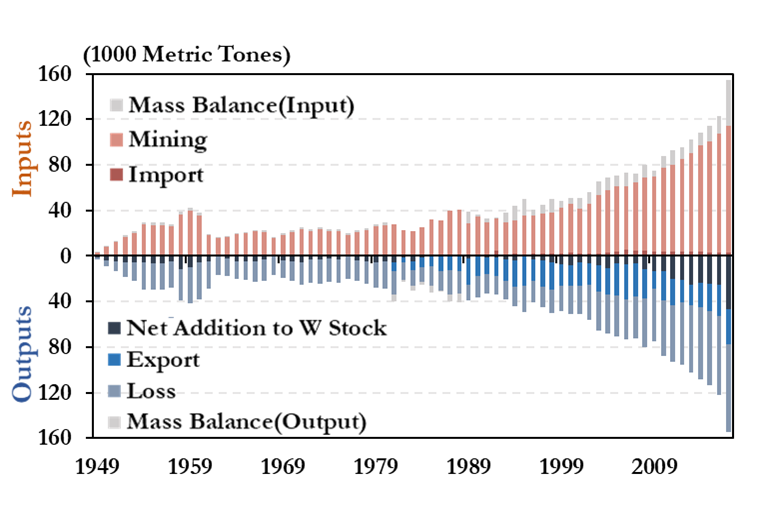


**Fig. S6 Mass balance of tungsten in China from 1949 to 2017**

| **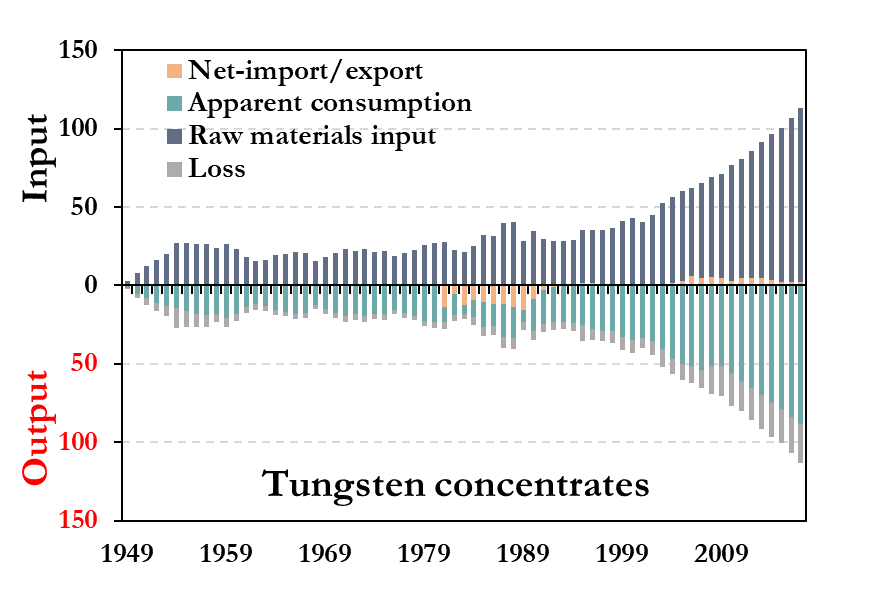** | **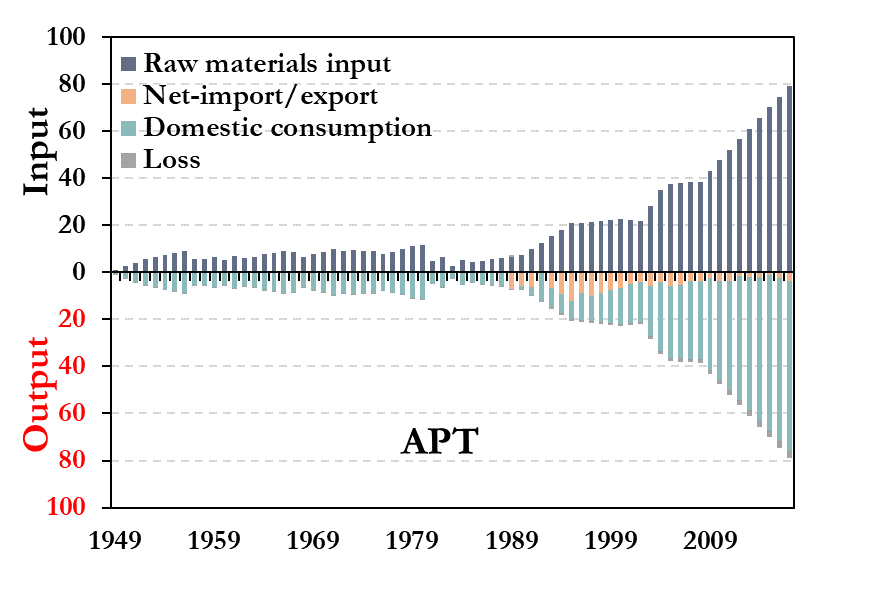** |
| --- | --- |
| **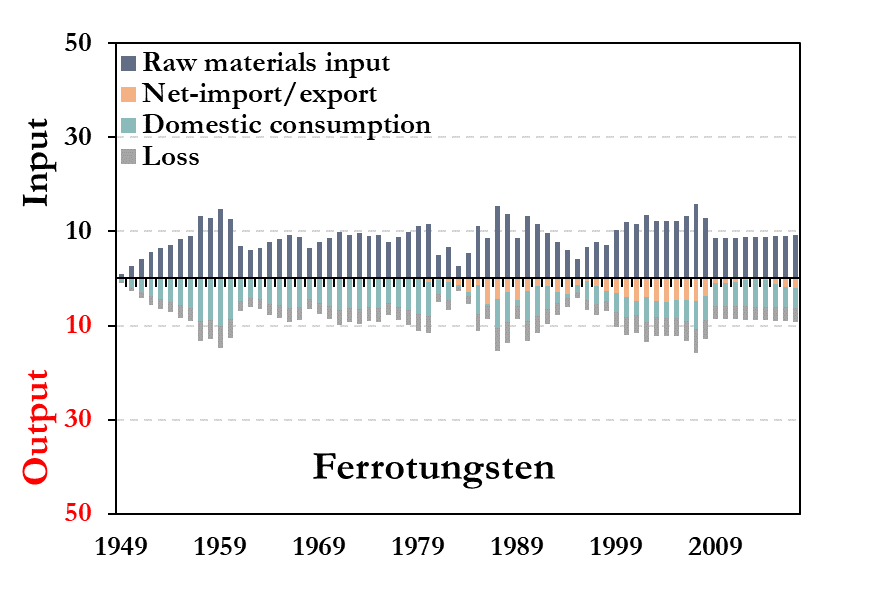** | **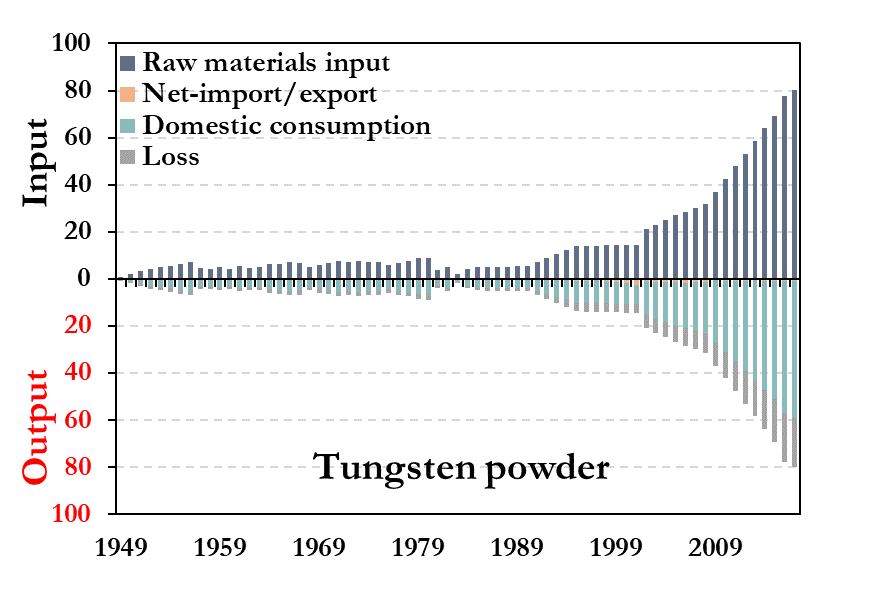** |
| **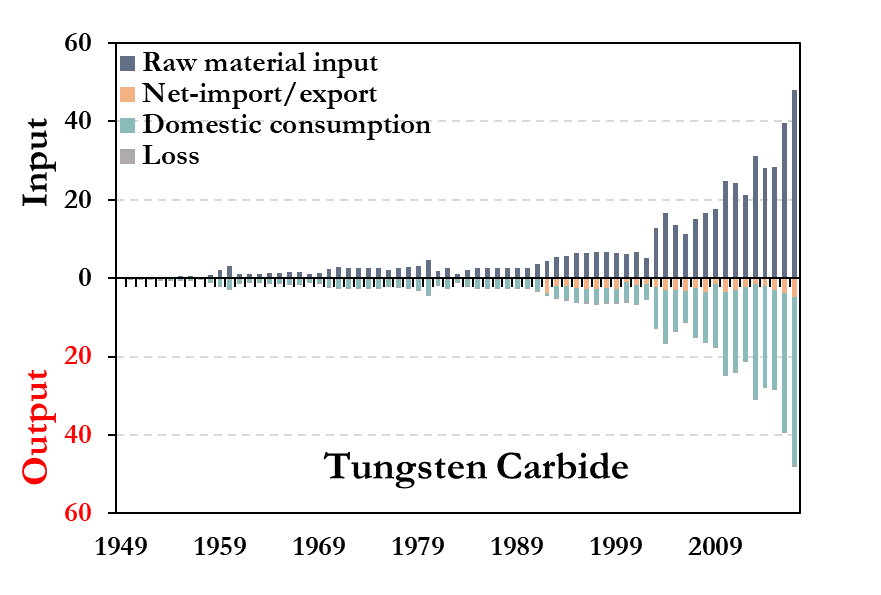** | **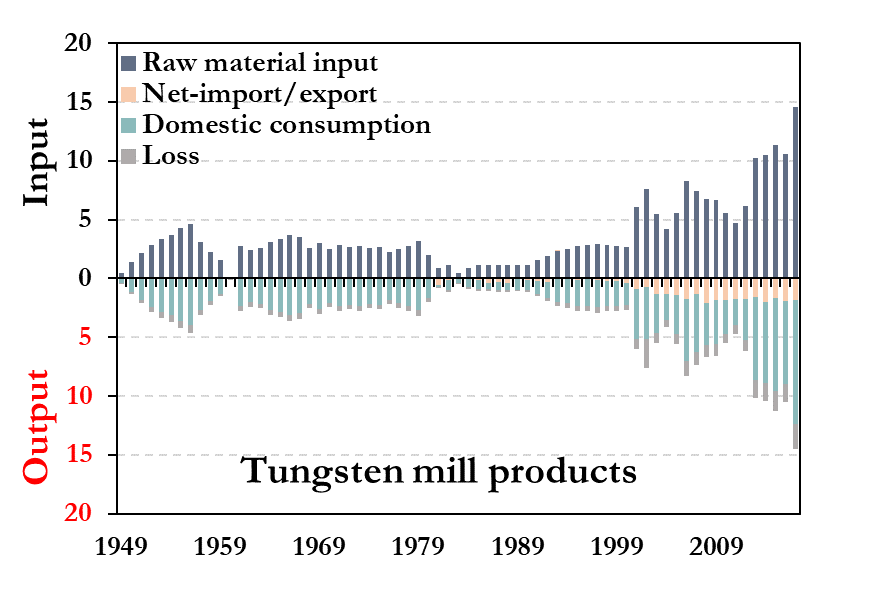** |
| **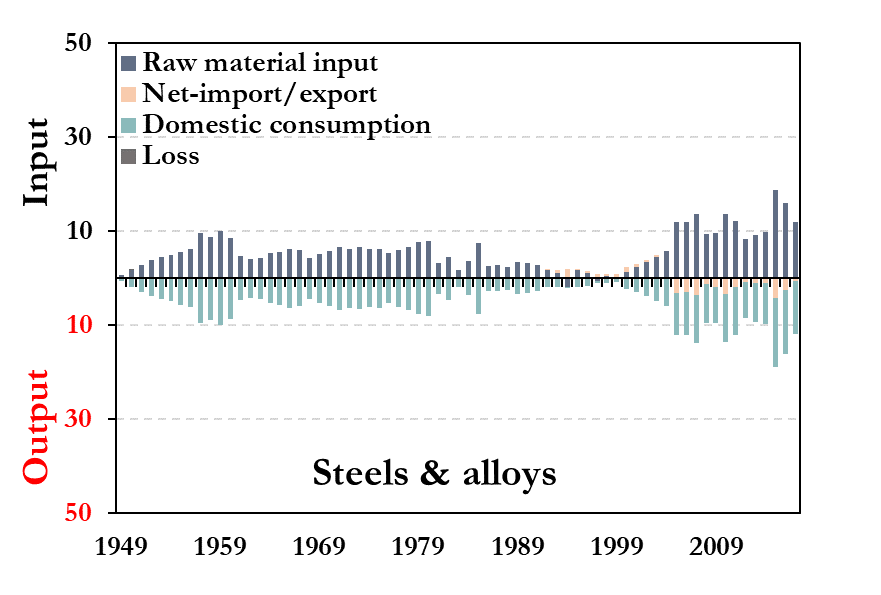** | **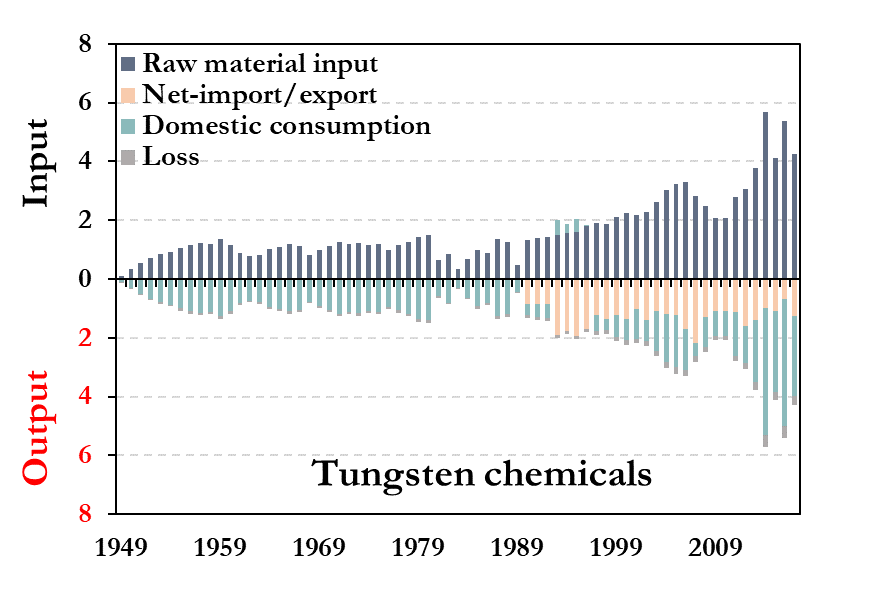** |

**Fig. S7 Mass balance of tungsten products in China from 1949 to 2017**

# References

Brunner, P.H., Rechberger, H., 2003. Practical Handbook of Material Flow Analysis. Lewis Publishers.

Christian, J., Singh Gaur, R.P., Wolfe, T., Trasorras, J.R.L., 2011. Tungsten Chemicals and their Applications. Tungsten 1–12. available at https://www.itia.info/news-2-title.html

China Nonferrous Metals Industry Association (CNMIA). The Yearbook of Nonferrous Metals Industry of China, Beijing. 1949-2017.

China Tungsten Industry Association (CTIA). The Yearbook of Tungsten Industry of China, Beingjing, 2017

Graedel, T.E., Harper, E.M., Nassar, N.T., Nuss, P., Reck, B.K., 2015. Criticality of metals and metalloids. Proc. Natl. Acad. Sci. 112, 4257–4262. https://doi.org/10.1073/pnas.1500415112

Gunn, G., 2014. Critical Metals Handbook, First. ed. John Wiley & Sons, Ltd, London.

Harper, E.M., 2008. A product-level approach to historical material flow analysis: Tungsten as a case study. J. Ind. Ecol. 12, 768–784. https://doi.org/10.1111/j.1530-9290.2008.00070.x

Harper, E.M., Graedel, T.E., 2008. Illuminating tungsten’s life cycle in the United States: 1975-2000. Environ. Sci. Technol. 42, 3835–3842. https://doi.org/10.1021/es070646s

Jones, N., Specialist, E., Aerofoils, T., 2017. Tungsten in Superalloys. ITIA Newsl. 1–20. available at https://www.itia.info/news-2-title.html

Leal-Ayala, D.R., Allwood, J.M., Petavratzi, E., Brown, T.J., Gunn, G., 2015. Mapping the global flow of tungsten to identify key material efficiency and supply security opportunities. Resour. Conserv. Recycl. 103, 19–28. https://doi.org/10.1016/j.resconrec.2015.07.003

Li, Z., 2005. Strategic Analysis for Development of High Speed Steel in China. Iron Steel 001, 1–7.

Mitchell, P., 2010. Tungsten in Life and Medicine. ITIA Tungsten 1–14. available at https://www.itia.info/news-2-title.html

Moll, M.A., 2016. First- and End Use of Tungsten Annual Summary and Ten Year Forecast presented by.

United Nations, 2015. United Nations International Trade Statistics Harmonized Commodity Description and Coding System(HS).

Schubert, W.D., Lassner, E., Bohlke, W., 2010. Cemented carbides-a success story. Tungsten, Int., Tungsten Ind. Assoc. (ITIA)(June 2010) 12. available at https://www.itia.info/news-2-title.html

Stafford, P.T., 1988. Tungsten, in: Material Facts and Problems. pp. 881–894.

USGS, 2005. Tungsten Recycling in the United States in 2000. Reston, Virginia.

Website, C., n.d. China Bulk Commodity website. Shanxi Linkknow Technol. Co.,Ltd. URL http://www.cbcie.com/w/index.html

Zeiler, B., Schubert, W., Bartl, A., Wien, T.U., 2018. Recycling of Tungsten-Current share,economic limitation and future potential. available at https://www.itia.info/news-2-title.html

Zhu，Xiusheng, 2018. Expert Interview.
